# 2D Strategy for the Construction of an Enzyme-Activated NIR Fluorophore Suitable for the Visual Sensing and Profiling of Homologous Nitroreductases from Various Bacterial Species

**DOI:** 10.1021/acssensors.1c01216

**Published:** 2021-09-01

**Authors:** Tao Liu, Yifei Wang, Lei Feng, Xiangge Tian, Jingnan Cui, Zhenlong Yu, Chao Wang, Baojing Zhang, Tony D. James, Xiaochi Ma

**Affiliations:** † Dalian Key Laboratory of Metabolic Target Characterization and Traditional Chinese Medicine Intervention, College of Pharmacy, Dalian Medical University, Dalian 116044, China; ‡ Jiangsu Key Laboratory of New Drug Research and Clinical Pharmacy, Xuzhou Medical University, Xuzhou 221004, China; § State Key Laboratory of Fine Chemicals, Dalian University of Technology, Dalian 116024, China; ∥ Department of Chemistry, University of Bath, Bath BA2 7AY, U.K.; ⊥ School of Chemistry and Chemical Engineering, Henan Normal University, Xinxiang 453007, China

**Keywords:** nitroreductases, fluorescent probe, bacteria, visual sensing, protein identification

## Abstract

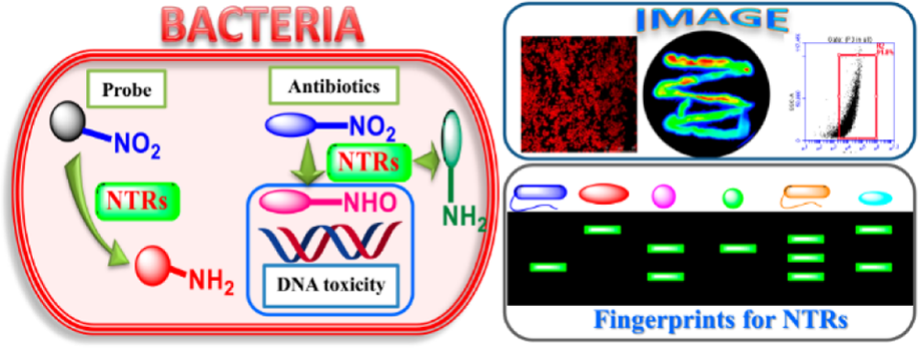

Nitroreductases (NTRs) mediate the reduction of nitroaromatic compounds to the corresponding nitrite, hydroxylamine, or amino derivatives. The activity of NTRs in bacteria facilitates the metabolic activation and antibacterial activity of 5-nitroimidazoles. Therefore, NTR activity correlates with the drug susceptibility and resistance of pathogenic bacteria. As such, it is important to develop a rapid and visual assay for the real-time sensing of bacterial NTRs for the evaluation and development of antibiotics. Herein, an activatable near-infrared fluorescent probe (**HC–NO**_**2**_) derived from a hemicyanine fluorophore was designed and developed based on two evaluation factors, including the calculated partition coefficient (Clog *P*) and fluorescence wavelength. Using **HC–NO**_**2**_ as the special substrate of NTRs, NTR activity can be assayed efficiently, and then, bacteria can be imaged based on the detection of NTRs. More importantly, a sensitive in-gel assay using **HC–NO**_**2**_ has been developed to selectively identify NTRs and sensitively determine NTR activity. Using the in-gel assay, NTRs from various bacterial species have been profiled visually from the “fluorescence fingerprints”, which facilitates the rapid identification of NTRs from bacterial lysates. Thus, various homologous NTRs were identified from three metronidazole-susceptible bacterial species as well as seven unsusceptible species, which were confirmed by the whole-genome sequence. As such, the evaluation of NTRs from different bacterial species should help improve the rational usage of 5-nitroimidazole drugs as antibiotics.

Nitroreductases (NTRs) are biological enzymes of the flavin enzyme family that reduce nitroaromatic compounds to the corresponding nitrite, hydroxylamine, or amino derivatives using nicotinamide adenine dinucleotide (NADH) or nicotinamide adenine dinucleotide phosphate (NADPH) as an electron donor.^1−9^ The hypoxic environment of the tumor tissue results in the overexpression of NTRs in tumor cells, highlighting the importance of NTR monitoring for clinical diagnosis and tumor therapy.^10,11^ Compared with the role of NTRs in mammalian cells, bacterial NTRs are thought to play a vital role in the antibacterial activity of nitroimidazole antibiotics, such as chloramphenicol.^12−17^ In bacterial cells, intracellular reduction of the nitro groups of 5-nitroimidazole drugs (metronidazole, tinidazole, and ornidazole) can be mediated by endogenous NTRs along with the production of active radical intermediates, which inhibits bacterial colonization through the inhibition of DNA synthesis. Clinically, emerging problems of resistance to 5-nitroimidazole drugs make the treatment of bacterial infections a growing challenge.^18^ As such, more and more metronidazole resistance has been reported around the world.^19,20^ Gene mutations of NTRs in various bacteria are thought to be correlated with 5-nitroimidazole susceptibility.^21−24^ Thus, the characterization of homologous NTRs for various clinically isolated pathogenic bacterial strains and mutant bacteria with 5-nitroimidazole resistance is important for evaluating drug susceptibility and treatment of bacterial infection. In addition, the existence of NTRs in bacterial species has resulted in the development of novel antibacterial agents based on drug release activated by endogenous bacterial NTRs.^25−27^ Therefore, the expression and bioactivity of NTRs in various pathogenic bacterial species are essential for clinical infection therapy, for which an efficient analytic technique is required for endogenous bacterial NTR profiling and identification.

Based on the reduction function of NTRs, fluorescent probes with a nitro group as the triggering moiety have been developed and used to detect mammalian NTRs in cancer cells under a hypoxic environment, facilitating their application in the diagnosis and therapy of cancer.^28−47^ For bacterial NTRs, although some fluorescent probes have been synthesized,^44,48−60^ suboptimal biocompatibility and photo-physicochemical properties were observed. In addition, distinct endogenous bacterial NTRs have not previously been visually profiled to assess their activity and metronidazole susceptibility.

With the present research, two evaluation factors were used to help determine appropriate fluorescent probes for NTRs. The two factors used were Clog *P* (which is related to solubility and permeability) and the fluorescence emission wavelength. Using this approach, we developed a near-infrared (NIR) fluorescent probe **HC–NO**_**2**_ derived from a hemicyanine fluorophore for sensing bacterial NTRs. Furthermore, using **HC–NO**_**2**_ as a staining dye for native polyacrylamide gel electrophoresis (PAGE), bacterial NTRs could be profiled visually, which both facilitated the efficient identification of bacterial NTRs and established a fingerprint of the NTRs for bacterial species.

## Results and Discussion

### Fluorophore Design Using Two Factors

A well-designed biological molecule should possess good biocompatibility, such as solubility in a physiological environment and membrane permeability. According to the “drug-likeness” rule, log *P* (where *P* is the partition coefficient) is closely related with the biocompatibility of a molecule. As such, biological molecules with Clog *P* over a range from 1 to 4 exhibit sufficient lipid affinity to cross membrane barriers and adequate water solubility to diffuse and dissolve in body fluids.^61^ Therefore, with the current research, Clog *P* was calculated for previously reported NTR fluorescent probes.

In addition, appropriate photo-physicochemical properties are key factors for fluorescent probe development. In particular, NIR fluorescent probes have the advantage of minimum interference from background fluorescence, result in the minimum photodamage, and have consequently been extensively used for the real-time imaging of cells, tissues, and live systems.^62−65^ Thus, the fluorescence spectral characteristics for previous NTR fluorescent probes have been collated to help guide the choice of an appropriate target NIR probe.

**Figure 1:**
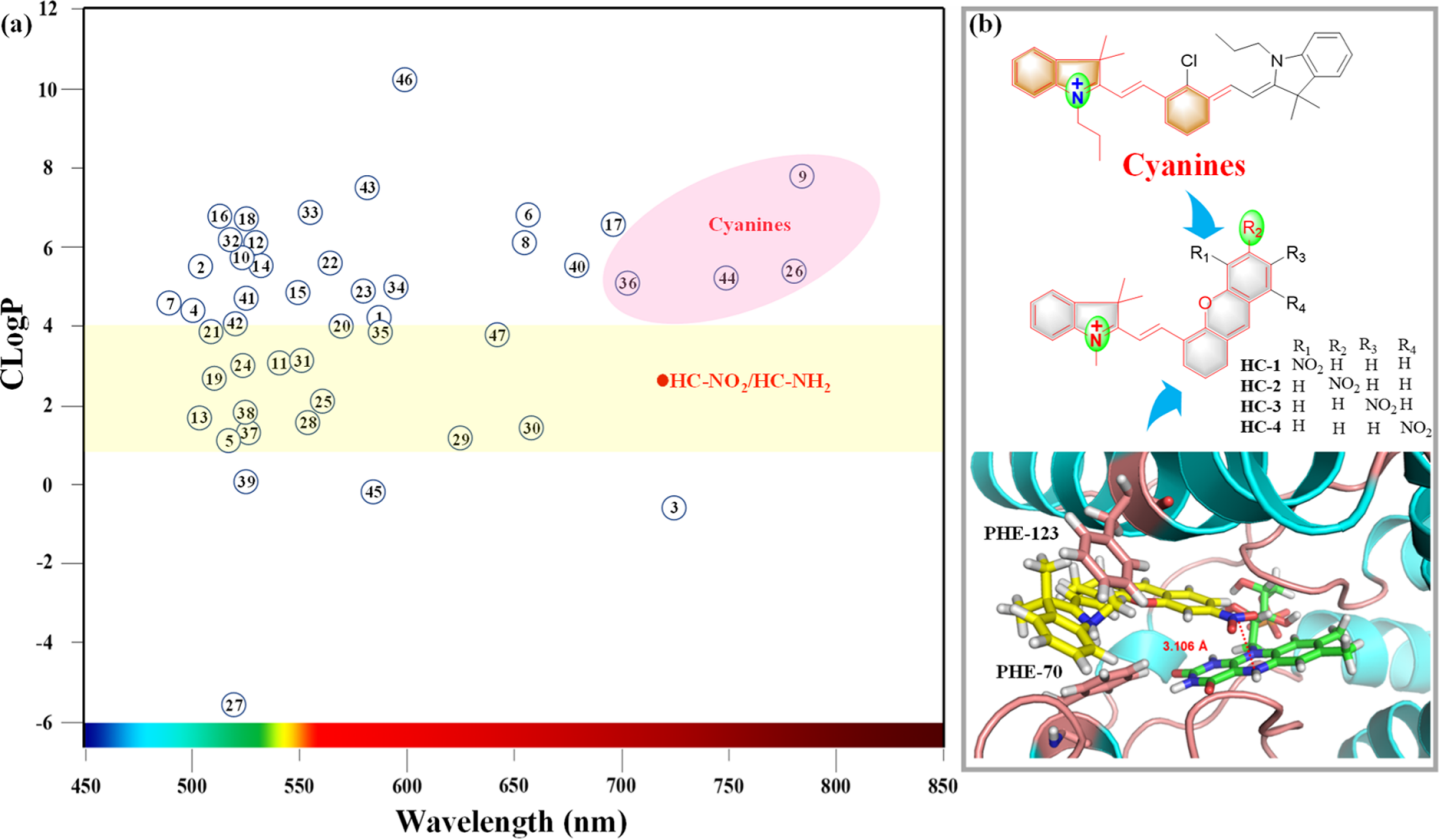
(a) Correlation analysis for Clog *P* and λ_em_ for previously reported fluorescent molecules and target probe **HC–NO**_**2**_**/HC–NH**_**2**_ for NTRs. (b) Illustration of **HC–NO**_**2**_ derived from the cyanine skeleton and based on the docking analysis of **HC–NO**_**2**_ and NTRs.

As such, we correlated Clog *P* and the fluorescence emission wavelength of 46 available fluorescent probes for NTRs (Table S1). As shown in Figure 1a, about 20 NTR fluorescent probes exhibited a suitable partition coefficient (Clog *P* of 1–4). However, most of them exhibited short fluorescence emission wavelengths. However, probes **3**, **9**, **26**, **36**, and **44** exhibited NIR fluorescence emissions of more than 700 nm. Among these probes, **9**, **26**, **36**, and **44** are derived from a cyanine fluorophore, which was suggestive of a suitable NIR fluorophore skeleton for our work. However, these probes exhibited undesirable Clog *P* values (>5), with hemicyanine (**36**) having the smallest Clog *P* value of 5.16.

Based on these two evaluation factors, hemicyanine was chosen as a suitable NIR fluorescent unit for NTRs. We then set about improving the biocompatibility. First, a methyl moiety was used instead of the ethyl group for the quaternary ammonium N atom to improve the water solubility and lower Clog *P*. Second, a nitro group was added as a substituent to the aromatic ring as a recognition moiety for NTRs. Therefore, four nitro-substituted hemicyanine analogues were synthesized (Figure 1b). According to the enzymatic reduction by NTRs, compound **HC-2** (**HC–NO**_**2**_) could be reduced to the amino form, while compounds **HC-1**, **HC-3**, and **HC-4** were unsuitable substrates for NTRs. Furthermore, in silico docking was performed to evaluate the interaction between the hemicyanine analogues and NTRs (Figures 1b and S1). The benzopyrrole moiety of the hemicyanine skeleton could dock with the PHE-70 and PHE-123 residues, resulting in the formation of a “sandwich” structure, and compound **HC-2** exhibited the smallest distance between the N5-FMN of the NTR and the nitro group, indicating that compound **HC-2** with a nitro group at the para-position of the conjugated system was a good substrate for the NTR.

As such, the target fluorescent probe (**HC–NO**_**2**_) was developed, consisting of a hemicyanine dye with an ideal Clog *P* value (2.62). In addition, the reduced form of **HC–NO**_**2**_ with an amino moiety (**HC–NH**_**2**_) was expected to be an NIR fluorescent molecule (λ_em_ > 700 nm).^40^

### Enzyme-Activatable Fluorescent Probe HC–NO_2_ for NTR Detection

As described above, a nitro group was attached to a hemicyanine fluorophore skeleton, affording the fluorescent probe **HC–NO**_**2**_. Similarly, **HC–NH**_**2**_ possessing an amino group was prepared as the reduced product of **HC–NO**_**2**_. Compared with **HC–NO**_**2**_, a significant absorbance at 670 nm was observed for **HC–NH**_**2**_. When excited by a laser with wavelengths ranging from 600 to 670 nm, a strong fluorescence emission was observed (λ_max_ = 720 nm, Φ = 0.041) for **HC–NH**_**2**_; in comparison, minimal fluorescence intensity was observed for **HC–NO**_**2**_ (Φ = 0.004) when excited at 670 nm (Figure S2). These observations indicate that **HC–NO**_**2**_ could serve as a potential off–on NIR fluorescent probe for NTRs.

**Figure 2:**
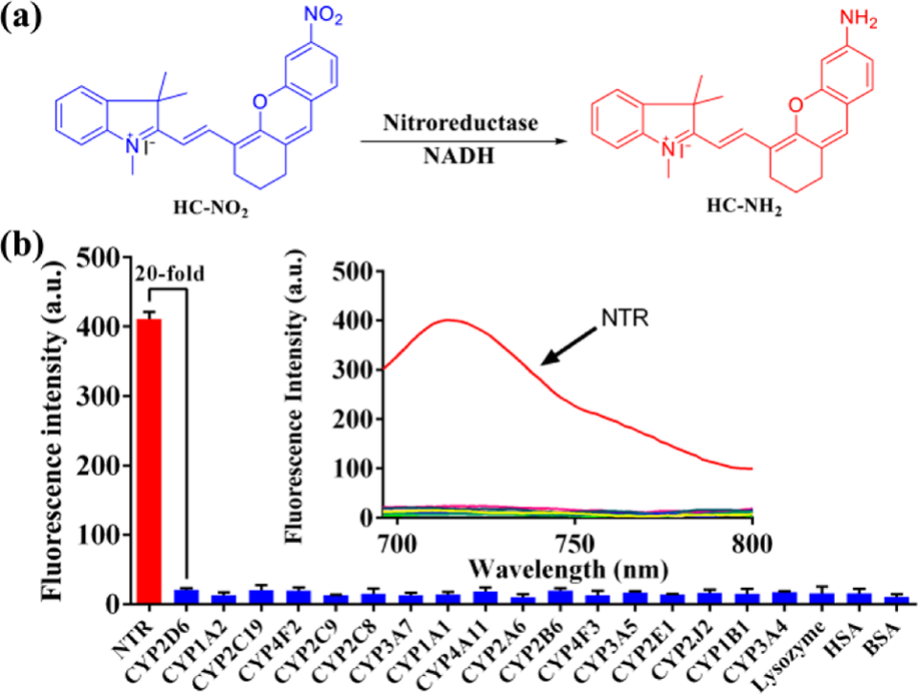
(a) Illustration of the reduction of **HC–NO**_**2**_ mediated by NTRs in the presence of NADH. (b) Fluorescence behavior of **HC–NO**_**2**_ toward various biological proteins in comparison with that toward NTRs.

Based on the biological function of NTRs, an enzymatic reduction of **HC–NO**_**2**_ is expected (Figure 2a). In our work, the coincubation of **HC–NO**_**2**_ and NTRs in the presence of NADH was analyzed using high-performance liquid chromatography (HPLC), where a peak corresponding to **HC–NH**_**2**_ was observed, indicating the enzymatic reduction and production of **HC–NH**_**2**_ (Figure S3). Furthermore, menadione, a known inhibitor for NTRs, was coincubated with **HC–NO**_**2**_ and NTRs, and a smaller chromatographic peak was observed for **HC–NH**_**2**_.^53^ Therefore, NTRs could mediate the reduction of **HC–NO**_**2**_ in the presence of NADH with **HC–NH**_**2**_ as the product.

As a potential fluorescent probe for NTRs, the fluorescence intensities of **HC–NO**_**2**_ and **HC–NH**_**2**_ in phosphate-buffered saline (PBS) with different pH values were evaluated. **HC–NO**_**2**_ exhibited no fluorescence at any pH, while **HC−NH**_**2**_ exhibited strong fluorescence intensity over a range of pH from 4 to 9 (Figure S4). Similarly, the fluorescence intensity induced by the production of **HC–NH**_**2**_ dependent on the reductase activity of NTRs has been evaluated in various solutions over a pH range from 2 to 12. Strong fluorescence was observed in solutions over a pH range from 6 to 8, with the strongest intensity at pH 7, which indicated that this was the most suitable incubation conditions for the strongest reductase activity of NTRs. Finally, in consideration of the use of **HC–NO**_**2**_ in a physiological environment (*e.g.*, bacteria), the optimal coincubation conditions for enzymatic reduction were determined to be pH 7.4 and 37 °C (Figures S5 and S6). For a certain concentration of **HC–NO**_**2**_ (10 μM), a concentration gradient of the NTR was used to evaluate the fluorescence responses, affording successive fluorescence spectra (Figure S7). A good linear relationship was obtained between the fluorescence intensity and concentration of the NTR (0–0.5 μg/mL), indicating potential application for an NTR activity quantitative assay. Furthermore, a quick enzymatic reaction was observed due to an excellent linear relationship between the fluorescence intensity and incubation time (0–5 min) (Figure S8). The kinetics for the enzymatic reduction of **HC–NO**_**2**_ by NTRs was evaluated using Michaelis–Menten kinetics (*V*_max_ = 387.2 nmol/min/mg, *K*_m_ = 17.87 μM) (Figure S9). To evaluate the specificity and selectivity of **HC–NO**_**2**_ toward NTRs, the reaction was evaluated in the presence of various species, including ions, amino acids, oxidizing agents, and reductive agents (Figures S10 and S11). Significantly, **HC–NO**_**2**_ exhibited good NTR specificity with no fluorescence response toward other species (Figure 2b), clearly indicating that **HC–NO**_**2**_ was a sensitive and selective fluorescent probe for NTRs.

### Sensing of Endogenous Bacterial NTRs and Imaging of Bacteria Using HC–NO_2_

**Figure 3:**
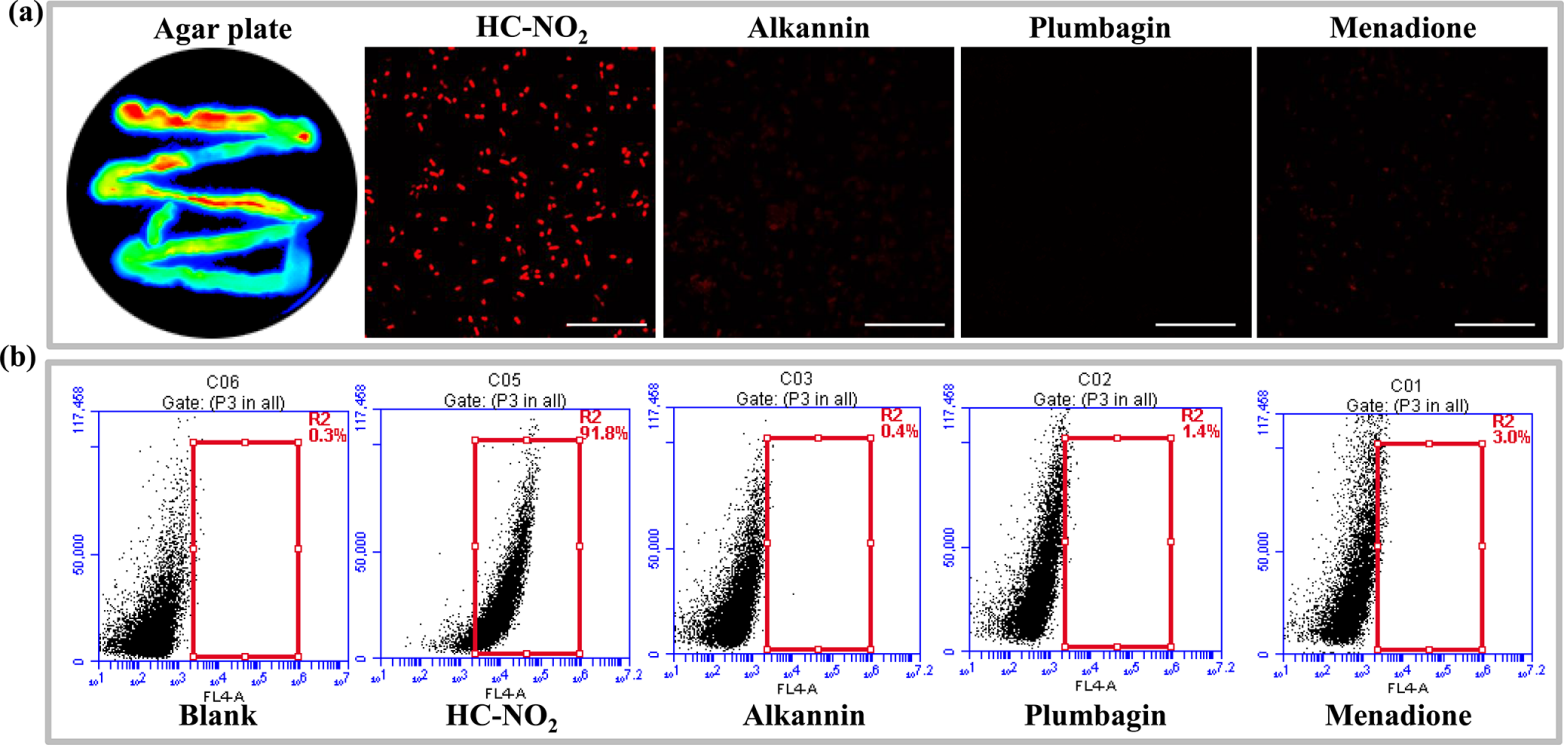
(a) Fluorescence images of *E. coli* 3079 on an agar plate together with CLSM images in the presence of inhibitors. Scale bar: 25 μm. (b) Flow cytometric analysis of *E. coli* 3079 stained using HC-NO_2_ in the presence of NTR inhibitors. Flow cytometric graph: (1) blank group, (2) control group, (3) alkannin, (4) plumbagin, and (5) menadione.

The fluorescent probe **HC–NO**_**2**_ was then used to monitor endogenous NTRs from various bacteria, including aerobic bacteria and facultative anaerobes (*Escherichia coli* 0377, *Streptococcus lactis*, *Streptococcus haemolyticus*, and *Lactobacillus salivarius*). **HC–NO**_**2**_ and **HC–NH**_**2**_ exhibited weak inhibition toward various bacterial species with an MIC (minimum inhibitory concentration) greater than 100 μM. After the coincubation of **HC–NO**_**2**_ and bacterial cells, the cells were imaged using a confocal microscope. As such, the bacterial cells were imaged successfully and endogenous NTRs could be detected by **HC–NO**_**2**_ (Figures 3a and S12). Agar plates are the main media used for the culture of bacterial colonies. Therefore, the fluorescent probe **HC–NO**_**2**_ was also used for the successful staining of bacterial colonies on agar plates, indicating the wide applicability of the **HC–NO**_**2**_ fluorescent probe (Figures 3a and S13). In addition, the production of **HC–NH**_**2**_ was confirmed in the bacterial culture using HPLC with a diode array detector (Figure S14). Then, using **HC–NO**_**2**_ as the substrate for an NTR activity assay, dicoumarol (IC_50_ 2.1 mM), menadione (IC_50_ 51.4 μM), plumbagin (IC_50_ 124.4 μM), and alkannin (IC_50_ 37.5 μM) displayed significant inhibitory effects on NTRs (Figure S15).

To confirm the NTR dependence of bacterial imaging by **HC–NO**_**2**_, the NTR inhibitors were added into the cultures of the bacteria. For the fluorescence imaging of *E. coli* 3079 and *Enterococcus faecalis*, weaker fluorescence signals were observed for the inhibitor groups in comparison with that of the control groups (Figures 3a and S16). Furthermore, flow cytometric analysis was performed, which confirmed the inhibitory effects based on the fluorescence signal (Figures 3b and S16). Therefore, **HC–NO**_**2**_ is an effective off–on fluorescent probe for bacterial NTR sensing, as evaluated using multiple imaging experiments.

### NTR Sensing of Anaerobic Bacteria with Metronidazole Susceptibility

**Figure 4:**
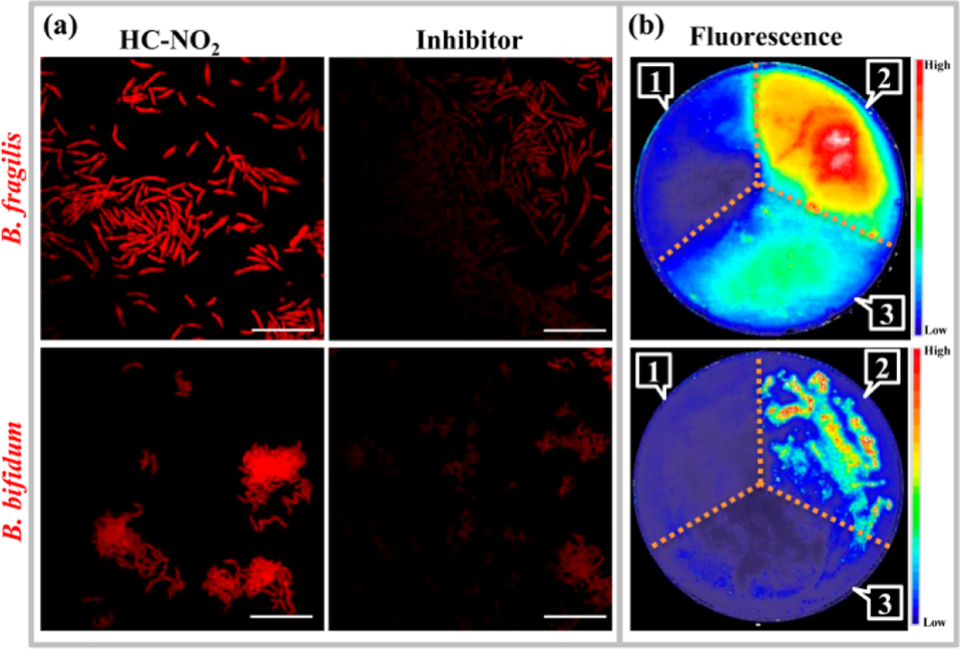
Fluorescence images of anaerobic bacterial species. (a) CLSM images of bacterial cells stained by **HC–NO**_**2**_ in the presence of the NTR inhibitor menadione (scale bar: 20 μm). (b) Fluorescence images of bacterial colonies on agar plates stained by **HC–NO**_**2**_. (1) Blank. (2) **HC–NO**_**2**_. (3) Menadione.

In contrast to the above aerobic bacteria and facultative anaerobes, three anaerobic bacterial strains *Bacteroides fragilis*, *Bacteroides thetaiotaomicron*, and *Bifidobacterium bifidum* were determined as being metronidazole-susceptible with MIC values of 0.5, 1, and 1 μg/mL, respectively. It is known that metronidazole is activated by endogenous NTRs with the intermediate possessing DNA toxicity, which could inhibit bacterial growth.^12−17^ Therefore, the NTRs expressed in bacteria need to be sensed and identified in order to assess the metronidazole susceptibility. After the coincubation of anaerobic bacterial strains and **HC–NO**_**2**_, the bacterial cells were imaged by confocal laser scanning microscopy (CLSM) based on the production of **HC–NH**_**2**_. As a result, red fluorescence images were obtained for anaerobic bacterial species (Figure 4a). Furthermore, using menadione as an NTR inhibitor, fluorescence images were measured for the bacterial cells, and weaker fluorescence intensities were observed. These results indicate that the NTR expressed by anaerobic bacteria *B. fragilis* and *B. bifidum* could be successfully detected in real time using **HC–NO**_**2**_. In addition to the bacterial cells in a liquid culture medium, bacterial colonies on solid agar plate supports are commonly evaluated for microbiological research. As such, the **HC–NO**_**2**_ probe was used to monitor the NTR from bacterial colonies on agar plates. The anaerobic bacterial colonies were cultured on agar plates and then divided into three areas corresponding to blank, **HC–NO**_**2**_, and inhibitor (menadione) areas. After imaging using a fluorescence scanner, distinct fluorescence signals were observed for different areas on the agar plate (Figures 4b and S17). Compared with the blank areas, the fluorescent probe areas displayed the strongest fluorescence signal, and weak fluorescence was observed for the areas with the inhibitor, indicating that the fluorescence imaging was NTR-dependent. Significantly, based on these fluorescence images, the expressions of NTRs for anaerobic bacterial species with metronidazole susceptibility could be determined.

### Sensitive Native Gel Assay for NTR Activity

**Figure 5:**
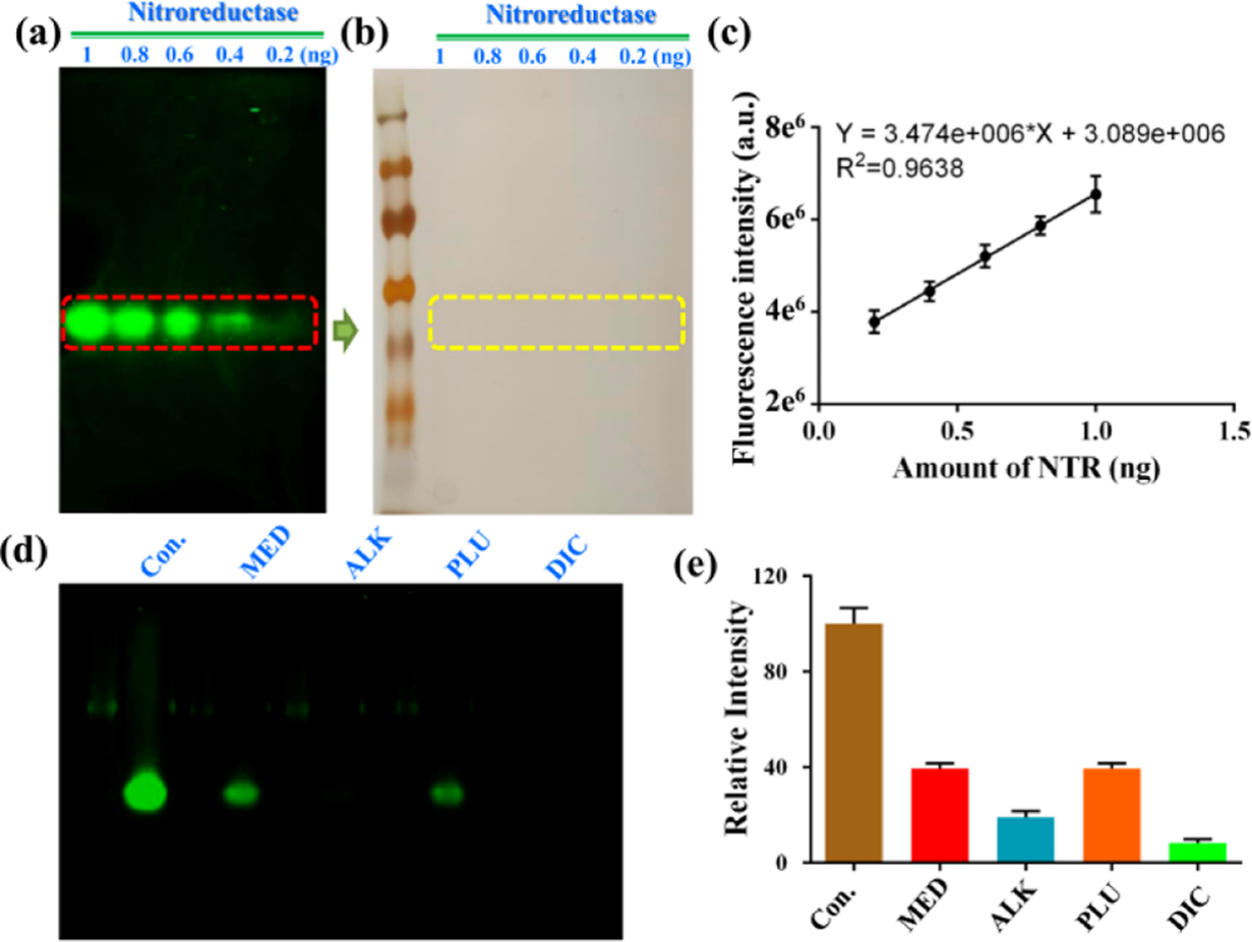
Native PAGE for NTRs stained using **HC–NO**_**2**_. Images of native PAGE with different loading amounts stained using (a) **HC–NO**_**2**_ and (b) silver. (c) Linear relationship between the fluorescence intensity of fluorescence bands on native PAGE and loading amounts of NTRs. (d) Inhibitory effects of NTR inhibitors on the gel and fluorescence intensity determination (e): **MED** (menadione), **ALK** (alkannin), **PLU** (plumbagin), and **DIC** (dicoumarol).

For the visual analysis of target proteins, the western blot is a generally used technique, which needs a special antibody for the target protein. However, for the molecular biological research into bacteria, the shortage of appropriate antibodies for bacterial proteins restricts the usage of the western blot. With our present research, **HC–NO**_**2**_ as an enzyme-activatable fluorescent probe can not only sense NTR selectively but also assay its activity. Therefore, using **HC–NO**_**2**_ as the staining reagent, we developed a visual native gel assay for NTR activity. The technique was established using native PAGE, keeping the biological activity of the loaded protein. Different loading amounts of the NTR were added to the native gel, and electrophoresis was performed using an ice-water bath to maintain the biological activity. Then, the gel was soaked in **HC–NO**_**2**_ PBS for enzymatic reduction. Using a fluorescence scanner, the gel was imaged, and the fluorescence bands resulting from **HC–NH**_**2**_, corresponding to the presence of NTR protein, were observed (Figure 5a). From the fluorescence image of the native gel assay of NTR activity, distinct fluorescence bands can be observed for an NTR loading of above 0.4 ng using the naked eye. However, the fluorescence intensity of the fluorescence bands corresponding to 0.2 ng of NTR can be determined using a fluorescence scanner. As such, the detection limit was determined to be approximately 0.4 ng, indicating a sensitive imaging method. Importantly, there was no band on the gel at the same loading when stained using the standard silver method (Figure 5b). The detection limit for the NTR stained using the known silver method was determined to be 30 ng (Figure S18). Furthermore, the fluorescence intensity of each band was determined, affording a good linear relationship with the NTR activity (Figure 5c). Thus, the native PAGE stained using **HC–NO**_**2**_ could detect NTR sensitively and determine NTR activity accurately. For the in-gel assay of the NTR, four inhibitors (menadione, alkannin, plumbagin, and dicoumarol) were used to inhibit the NTR activity prior to staining with **HC–NO**_**2**_. In the fluorescence images of the native gel, the lanes containing inhibitors exhibited significantly weaker fluorescence bands in comparison with the control lanes (Figure 5d). The fluorescence intensity determination also confirmed the inhibitory effect (Figure 5e). Based on these inhibitory experiments, the native gel assay for the NTR using **HC–NO**_**2**_ was reliable and exhibited potential for the evaluation of inhibitors.

### Visual Profiling of Homologous NTRs from Various Bacterial Species

**Figure 6:**
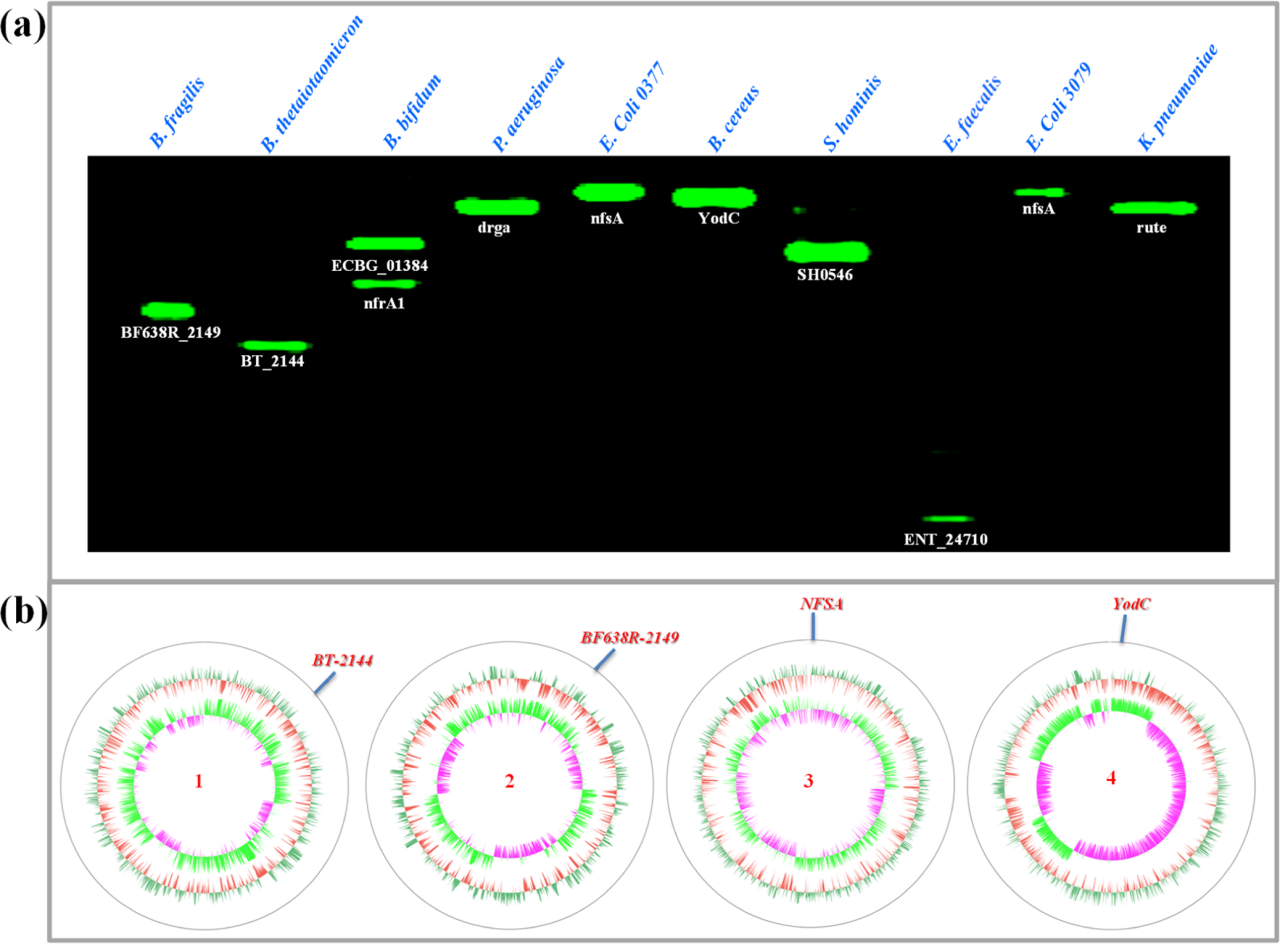
(a) Visual profiling of individual homologous NTRs from various bacterial species on the native gel stained using **HC–NO**_**2**_. (b) Homologous NTR expression confirmed from the genomes of the bacterial species [(1) *B. thetaiotaomicron*; (2) *B. fragilis*; (3) *E. coli* 0377; and (4) *B. cereus*].

Ten bacterial strains, including anaerobic bacteria (*B. fragilis*, *B. thetaiotaomicron*, and *B. bifidum*) and aerobic bacteria (*Pseudomonas aeruginosa*, *E. coli* 0377, *Bacillus cereus*, *Staphylococcus hominis*, *E. faecalis*, *E. coli* 3079, and *Klebsiella pneumoniae*), were evaluated for their susceptibility to metronidazole. Three anaerobic bacterial strains were significantly inhibited by metronidazole, with MICs ≤ 1 μg/mL (Table S2). However, the other seven aerobic bacterial strains were resistant to metronidazole (MICs > 64 μg/mL). As the key metabolic activatable enzyme for metronidazole, the expression of NTRs in these bacterial species attracted our interest. Using our in-gel assay, the individual NTRs from these bacterial species were then explored. The bacterial lysates were loaded into the gel, and electrophoresis was performed to obtain the separation of multiple proteins. **HC–NO**_**2**_ was used to detect the NTR activity. After the gel was run, a fluorescence image of the gel was obtained using a fluorescence scanner. As shown in Figure 6a, each bacterial species expressed active NTRs, as indicated by fluorescence bands. Among these fluorescence bands, the weakest fluorescence intensity was for the lane of *E. faecalis,* suggesting the lowest expression of NTRs. Most of the bacterial species exhibited single fluorescence bands, indicating the expression of one homologous NTR. However, two fluorescence bands were observed for the *B. bifidum* lysate, indicating the existence of two homologous NTRs. Among the 11 fluorescence bands on the gel, just two bands moved the same distance, indicating the same NTR protein for the lanes of *E. coli* 0377 and *E. coli* 3079, which were similar lab strains. Thus, the fluorescence image for the in-gel assay of bacterial lysates stained using **HC–NO**_**2**_ provided information about the number of bands, fluorescence intensity, and distance moved, which provided a profile for the NTRs of each bacterial species and established “fingerprints” for homologous NTRs in various bacterial species. As such, the fluorescent probe **HC–NO**_**2**_ could be used to efficiently image gels for protein analysis.

As mentioned above, the individual NTRs for various bacterial species could be discriminated selectively using the native gel assay. Subsequently, the fluorescence bands corresponding to the individual NTRs were excised and identified using mass spectrometric analysis. The genetic names of the homologous NTRs are given under the fluorescence bands of the gel and shown in Figure 6a. Accordingly, distinct homologous NTRs are expressed in various bacterial species, all of which could mediate the reduction of **HC–NO**_**2**_ to produce **HC–NH**_**2**_. However, the NTRs of the metronidazole unsusceptible bacterial species may mediate the reduction using a different mechanism. The homologous NTRs BF638R_2149, BT_2144, nfrA1, and ECBG_01384 were identified for metronidazole-susceptible bacteria *B. fragilis*, *B. thetaiotaomicron*, and *B. bifidum*, which have been proposed to transform metronidazole into an active intermediate exhibiting DNA toxicity. As such, the bacterial species exhibiting the expression of the above NTRs (BF638R_2149, BT_2144, nfrA1, and ECBG_01384) are metronidazole-susceptible and as such are suitable for clinical antibacterial treatment. Finally, the genomes of the four bacterial species *B. thetaiotaomicron*, *B. fragilis*, *E. coli* 0377, and *B. cereus* were sequenced, and the encoding genes for the NTRs were determined (Figure 6b).

## Conclusions

Using the two evaluation factors of Clog *P* and the fluorescence emission wavelength, a fluorescent probe (**HC–NO**_**2**_) derived from a cyanine fluorophore was developed, exhibiting “drug-like” Clog *P* (which indicates good biocompatibility) and NIR fluorescence emission. The developed fluorescent probe can be activated by NTRs in the presence of NADH. Based on enzymatic reduction, **HC–NO**_**2**_ was then used to assay NTR activity *in vitro* and monitor endogenous bacterial NTRs in addition to imaging bacteria *in vivo*. Using the enzymatic reduction of **HC–NO**_**2**_ as a staining method, a native gel assay was developed to visually monitor NTRs, which was more sensitive than the usual silver method. Importantly, by measuring the fluorescence intensity bands, the NTR activity could be accurately determined. Furthermore, the homologous NTRs were profiled visually for various bacterial species, along with rapid protein identification. Since NTRs are a key metabolic enzyme for metronidazole, the profiling of NTRs from metronidazole-susceptible bacterial species can indicate potential biomarkers for testing medicinal susceptibility in the future. Thus, the in-gel monitoring of NTRs not only facilitated fluorescence differentiation of bacterial species using “fingerprints” but also could be used to investigate metronidazole susceptibility and antibacterial treatments.

## Supporting Information Available

Apparatus and methods, synthesis and characterization of compounds, spectroscopy, fluorescence behavior of **HC–NO_2_**, and bioimaging data (PDF)
