# Supplementary material for: 2D Strategy for the Construction of an Enzyme-Activated NIR Fluorophore Suitable for the Visual Sensing and Profiling of Homologous Nitroreductases from Various Bacterial Species
Source: ACS Sens. 2021 Sep 1;6(9):3348–56. doi: 10.1021/acssensors.1c01216 (PMC8477384; doi:10.1021/acssensors.1c01216)
Supplement: Supplementary file 1 — se1c01216_si_001.pdf [file se1c01216_si_001.pdf]

Supporting Information

2D strategy for the construction of an enzyme activated NIR fluorophore suitable for the visual sensing and profiling of homologous nitroreductases from various bacterial species

Tao Liu,[†,§,#] Yifei Wang,[†,#] Lei Feng,[†,#] Xiangge Tian,[†] Jingnan Cui,[§] Zhenlong Yu,[†] Chao Wang,[†]* Baojing Zhang,[†] Tony D. James,[‖,※]* Xiaochi Ma[†,‡]*

[†]Dalian Key Laboratory of Metabolic Target Characterization and Traditional Chinese Medicine Intervention, College of Pharmacy, Dalian Medical University, Dalian 116044, China

[‡]Jiangsu Key Laboratory of New Drug Research and Clinical Pharmacy, Xuzhou Medical University, Xuzhou 221004, China.

[§]State Key Laboratory of Fine Chemicals, Dalian University of Technology, Dalian 116024, China

[‖]Department of Chemistry, University of Bath, Bath BA2 7AY, UK

[※]School of Chemistry and Chemical Engineering, Henan Normal University, Xinxiang 453007, China

[#] These authors contributed equally to this work.

***Corresponding Author**

Chao Wang − Dalian Key Laboratory of Metabolic Target Characterization and Traditional Chinese Medicine Intervention, College of Integrative Medicine, College of Pharmacy, Dalian 116044, China; orcid.org/0000-0002-6251-7908; Email: wach_edu@sina.com

Tony D. James –Department of Chemistry, University of Bath, Bath BA27AY, UK; orcid.org/0000-0002-4095-2191; Email: t.d.james@bath.ac.uk

Xiaochi Ma − Dalian Key Laboratory of Metabolic Target Characterization and Traditional Chinese Medicine Intervention, College of Integrative Medicine, College of Pharmacy, Dalian 116044, China; orcid.org/ 0000-0003-4397-537X; Email: maxc1978@163.com

Index

Experimental S3
Materials and apparatus S3
Synthesis of fluorescent probe HC-NO_2 .... S4
Scheme S1. Synthesis of fluorescent probe **HC-NO_2 (HC-2)** and its derivatives.. .... S4
Determination of the quantum yield (QY) S7
LogP (partition coefficient) calculation of fluorophores S7
Enzymatic reduction of HC-NO_2 mediated by nitroreductase (NTR) in the presence of nicotinamide adenine dinucleotide (NADH) S7
Investigation about inhibitory effects of compounds on NTR S8
Fluorescence imaging of bacterial cells by HC-NO_2 using confocal laser scanning microscopy S9
Fluorescence imaging of bacterial colonies by HC-NO_2 on agar plates S9
Native-PAGE electrophoresis for bacterial lysates and NTR proteins S9
Table S1. Calculated log P and excitation/emission wavelength of reported fluorophores. S11
Figure S1. Chemical structures of **HC-1** (a), **HC**-3 (b), **HC-4** (c) and *in silico* docking analysis between these molecules and NTR protein. S19
Figure S2. (a) The absorption spectra of **HC-NO_2** and **HC-NH_2**. (b) The fluorescence spectra of **HC-NO_2** and enzymatic reduction of **HC-NO_2** mediated by NTR. S19
Figure S3. HPLC chromatograms for the enzymatic reaction of **HC-NO_2** for the nitroreductase co-incubation culture.. .... S20
Figure S4. The effect of pH on the fluorescence intensity of **HC-NO_2** and **HC-NH_2** (10 μM) $\lambda_{ex.}$ 670 nm, $\lambda_{em.}$ 720 nm S20
Figure S5. The fluorescence intensities for the co-incubations of **HC-NO_2** and NTR in phosphate solution at different pH values ($\lambda_{ex.}$ 670 nm, $\lambda_{em.}$ 720 nm). S21
Figure S6. The fluorescence intensities for the co-incubations of **HC-NO_2** and NTR in phosphate solution at different temperature ($\lambda_{ex.}$ 670 nm, $\lambda_{em.}$ 720 nm). S21
Figure S7. (a) The fluorescence responses of **HC-NO_2** towards different NTR concentrations (0, 0.025, 0.05, 0.075, 0.1, 0.125, 0.15, 0.175, 0.2, 0.3, 0.4, 0.5, 1, 2, 3 μg/mL). (b) The relationship between the fluorescence intensity and NTR concentrations. S21
Figure S8. (a) The fluorescence responses of **HC-NO_2** towards NTR for different incubation times (0, 1, 2, 3, 4, 5, 6, 8, 10, 15, 20, 30, 60 min). (b) The relationship between the fluorescence intensity and incubation time S22
Figure S9. Kinetics about the enzymatic reduction of **HC-NO_2** mediated by NTR S22
Figure S10. (a) The fluorescence response of **HC-NO_2** towards various species (ions and amino acids). (b) The fluorescence intensity for the co-incubation of **HC-NO_2** and NTR in the presence of various species (ions and amino acids) S23
Figure S11. The fluorescence response of **HC-NO_2** towards various redox agents.. .... S23
Figure S12. Confocal laser scanning microscopic images of various general bacterial cells stained using **HC-NO_2**. S24
Figure S13. Fluorescence images of various general bacterial colonies on agar plates stained using **HC-NO_2** S24
Figure S14. HPLC chromatograms of **HC-NO_2** metabolites from *Escherichia coli* 4164 culture.. .... S25

Figure S15. The inhibitory effects of four inhibitors against NTR.. ....S25
Figure S16. (a) Confocal laser scanning microscopic images and (b) flow cytometric analysis of *E. faecalis* stained by **HC-NO$_2$** in the presence of inhibitors.. ....S26
Figure S17. (a) Bright image and (b) fluorescence image of *Bacteroides thetaiotaomicron* on an agar plate in the presence of **HC-NO$_2$** and inhibitor (menadione). S27
Figure S18. Sensitivity investigation for NTR assay on native PAGE-gel stained using silver. ..S27
Table S2. Inhibitory effect of metronidazole on various bacteria. S28
Figure S19. Total protein profiling of various bacteria species on native gel stained using Coomassie brilliant blue. S28
Figure S20. NTR expression of various bacteria species determined by native PAGE-gel using fluorescent probe **HC-NO$_2$** for staining. S29
Figure S21. ^{1}H NMR spectrum of compound **1** ($CDCl_3$). S30
Figure S22. ^{1}H NMR spectrum of **3-1** ($CDCl_3$). S30
Figure S23. ^{1}H NMR spectrum of **3-2** ($CDCl_3$). S31
Figure S24.^{1}H NMR spectrum of **3-3** ($CDCl_3$). S31
Figure S25.^{1}H NMR spectrum of **3-4** ($CDCl_3$). S32
Figure S26.^{1}H NMR spectrum of **HC-1** ($CDCl_3$). S32
Figure S27.^{13}C NMR spectrum of **HC-1** ($CDCl_3$). S33
Figure S28. High resolution mass spectrum of **HC-1**. S33
Figure S29. ^{1}H NMR spectrum of **HC-2** (**HC-NO$_2$**, $CDCl_3$). S34
Figure S30. ^{13}CNMR spectrum of **HC-2** (**HC-NO$_2$**, $CDCl_3$). S34
Figure S31. High resolution mass spectrum of **HC-2** (**HC-NO$_2$**). S35
Figure S32. ^{1}H NMR spectrum of **HC-3** ($CDCl_3$). S35
Figure S33. ^{13}CNMR spectrum of **HC-3** ($CDCl_3$) S36
Figure S34. High resolution mass spectrum of **HC-3**. S36
Figure S35. ^{1}H NMR spectrum of **HC-4** ($CDCl_3$). S37
Figure S36. ^{13}CNMR spectrum of **HC-4** ($CDCl_3$) S37
Figure S37. High resolution mass spectrum of **HC-4**. S38
Figure S38. ^{1}H NMR spectrum of **HC-NH$_2$** (MeOD). S38
References S38

Experimental

Materials and apparatus

All of the reagents for the preparation of bacterial culture medium such as agar, yeast extracts, glucose, tryptone, and NaCl were purchased from Dalian Meilun Biotechnology Co., Ltd (China). Chemical reagents for the synthesis of fluorescent probe were produced by Tianjin Kemio Chemical Reagent Co., Ltd (China). Chromatographic reagents, such as acetonitrile and methanol were purchased from sigma-aldrich (MERCK). Nitroreductase, lysozyme, bovine serum albumin (BSA), and

human albumin (HSA) were purchased from sigma-aldrich (MERCK). CYP450 enzymes were purchased from Corning Incorporated Life Sciences.

NMR spectra of the target molecule were acquired using a Bruker-500 with tetramethylsilane (TMS) as the internal standard (Bruker, USA). High resolution mass spectral (HRMS) analysis was performed using a G6224A TOF-MS. A constant temperature incubator shaker (HZQ-C) was obtained from Harbin Donglian Electronic Technology Development Co., LTD (China). A confocal laser scanning microscope was provided by Leica (German). The fluorescence spectra and fluorescence intensity was determined using a BioTek Synergy H1 microplate reader (BioTek, USA). The fluorescence imaging of native-PAGE gel stained by **HC-NO_2** was performed using an Amersham Typhoon RGB (GE, USA). A Biosciences Accuri C6 flow cytometer was provided by Becton, Dickinson and Company (USA).

Synthesis of fluorescent probe HC-NO_2

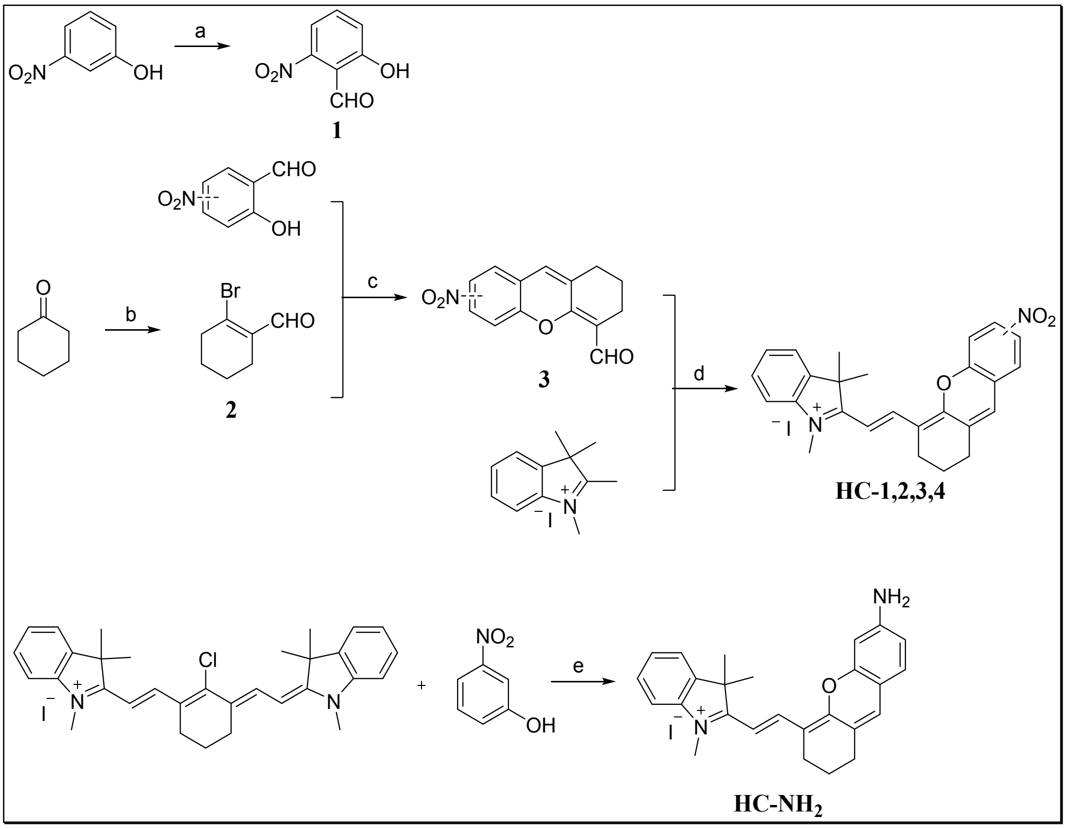

Scheme S1. Synthesis of fluorescent probe **HC-NO_2 (HC-2)** and its derivatives. Conditions: (a) HMTA, TFA; (b) PBr_3, DMF, $CHCl_3$; (c) Cs_2CO_3, DMF; (d) K_2CO_3, Ac_2O; (e) i) Et_3N, DMF; ii) $SnCl_2$, HCl, MeOH.

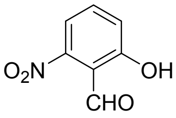

1

Compound 1: 3-Nitrophenol (2 g, 14 mmol) and HMTA (2.42 g, 17 mmol) were dissolved in TFA (10 mL) and the mixture was refluxed for 12 hours. After consumption the starting material, the reaction mixture was concentrated *in vacuo* and purified on silica gel (hexane/AcOEt: 10/1) to afford a white solid (440 mg, 19%). ^{1}H NMR (500 MHz, $CDCl_3$) δ 12.11 (s, 1H), 10.34 (s, 1H), 7.62 (d, J = 8.2 Hz, 1H), 7.56 (d, J = 7.8 Hz, 1H), 7.30 (d, J = 8.5 Hz, 1H).

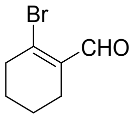

2

Compound 2: To a solution of DMF (22.4 mL, 290 mmol) in $CHCl_3$ (150 mL) at 0 °C PBr_3 was lowly added slowly dropwise (24.8 mL, 261 mmol). After 1 hour, cyclohexanone (10 mL, 96.8 mmol) was added and the mixture was stirred overnight at 25 °C. Then, the red solution was poured onto ice and pH adjusted to 7 using solid $NaHCO_3$. The organic layer was separated and the aqueous layer was extracted with DCM (150 mL×3). The organic layer was combined, and dried over Na_2SO_4, filtered and concentrated *in vacuo* to provide compound **2** as yellow oil (12.4 g). Without any purification, the yellow oil was direct used for the next step.

General procedure for compound 3: To a solution of nitro-salicylaldehyde (167 mg, 1 mmol) in DMF (10 mL), was added compound **2** (277 mg, 1.2 mmol) and Cs_2CO_3 (978mg, 3 mmol), which was stirred for 2 hours at room temperature. Then, the mixture was diluted in deionized water (50 mL) and extracted twice with AcOEt (50 mL×3); the organic layer was dried over Na_2SO_4, filtered and concentrated *in vacuo*. Purification of the crude residue on silica gel (hexane/AcOEt: 4/1) afforded **3-1, 3-2, 3-3** and **3- 4** as yellow solids.

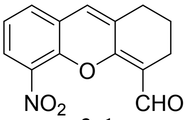

3-1

3-1： 162 mg, yield 63%. ^{1}H NMR (500 MHz, $CDCl_3$) δ 10.42 (s, 1H), 7.92 (dd, J = 8.3, 1.4 Hz, 1H), 7.37 (dd, J = 7.6, 1.4 Hz, 1H), 7.14 (t, J = 7.9 Hz, 1H), 6.67 (s, 1H), 2.65 – 2.59 (m, 2H), 2.47 (t, J = 6.0 Hz, 2H), 1.80 – 1.71 (m, 2H).

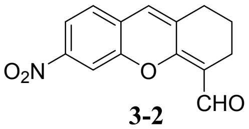

3-2

3-2: 146 mg, yield 57%. ^{1}H NMR (500 MHz, $CDCl_3$) δ 10.35 (s, 1H), 7.93 – 7.87 (m, 2H), 7.25 (d, J = 8.3 Hz, 1H), 6.67 (s, 1H), 2.61 (t, J = 6.0 Hz, 2H), 2.44 (t, J = 6.0 Hz, 2H), 1.77 – 1.69 (m, 2H).

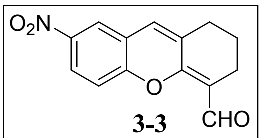

3-3

3-3: 152 mg, yield 59%. ^{1}H NMR (500 MHz, $CDCl_3$) δ 10.38 (s, 1H), 8.16 (d, J = 9.0 Hz, 1H), 8.06 (s, 1H), 7.18 (d, J = 9.0 Hz, 1H), 6.69 (s, 1H), 2.69 – 2.58 (m, 2H), 2.47 (t, J = 5.9 Hz, 2H), 1.81 – 1.71 (m, 2H).

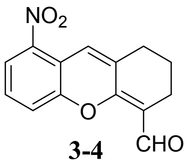

3-4

3-4: 178 mg, yield 69%. ^{1}H NMR (500 MHz, $CDCl_3$) δ 10.37 (s, 1H), 7.77 (d, J = 7.7 Hz, 1H), 7.43 (s, 1H), 7.40 – 7.33 (m, 2H), 2.70 – 2.64 (m, 2H), 2.47 (t, J = 6.0 Hz, 2H), 1.80 – 1.73 (m, 2H).

General procedure for HC-1, 2, 3, 4: To a solution of compound **3** (100 mg, 0.4 mmol) in Ac_2O (5 mL) was added 1,2,3,3-tetramethyl-3H-indolium iodide (144 mg, 0.48 mmol) and K_2CO_3 (111 mg, 0.8 mmol). After stirring for 12 h at 80 °C, a brick-red solution was obtained that was concentrated in vacuo. The residue was purified on silica gel (DCM /MeOH: 20/1) to afford **HC-1, 2, 3,** and **4** as dark brown solids.

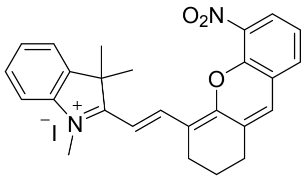

HC-1

HC-1: 65 mg, yield 30%. ^{1}H NMR (500 MHz, $CDCl_3$) δ 8.81 (d, J = 15.5 Hz, 1H), 8.13 (d, J = 7.2 Hz, 1H), 7.61 – 7.47 (m, 5H), 7.31 (d, J = 8.1 Hz, 1H), 7.06 (d, J = 15.5 Hz, 1H), 7.00 (s, 1H), 4.30 (s, 3H), 2.99 (t, J = 5.9 Hz, 2H), 2.78 – 2.71 (m, 2H), 2.03 – 1.95 (m, 2H), 1.88 (s, 6H). ^{13}C NMR (125 MHz, $CDCl_3$) δ 180.57, 156.55, 147.06, 146.08, 143.25, 141.73, 133.25 – 133.07, 132.57, 129.35, 128.81, 127.45, 126.53, 124.42, 123.93, 122.48, 117.66, 113.72, 110.30, 51.71, 36.09, 29.45, 27.27, 25.08, 19.98. HRMS (ESI+): *m/z* calcd for $C_{26}H_{25}N_2O_3^+$ $[M]^+$ 413.1860, found 413.1863.

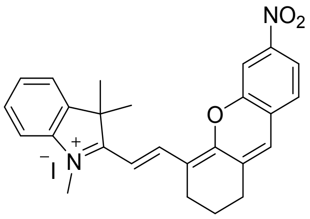

HC-2 (HC-NO$_2$)

HC-2 (HC-NO$_2$): 80 mg, yield 37%. ^{1}H NMR (500 MHz, $CDCl_3$) δ 8.60 (d, J = 15.5 Hz, 1H), 8.04 (dd, J = 8.4, 2.1 Hz, 1H), 7.97 (d, J = 2.0 Hz, 1H), 7.59 – 7.48 (m, 4H), 7.45 (d, J = 8.5 Hz, 1H), 7.09 (d, J = 15.5 Hz, 1H), 6.99 (s, 1H), 4.31 (s, 3H), 2.98 (t, J = 6.0 Hz, 2H), 2.75 (t, J = 5.7 Hz, 2H), 2.01 – 1.91 (m, 2H), 1.86 (s, 6H).^{13}C NMR (125 MHz, $CDCl_3$) δ 179.91, 157.49, 152.11, 148.10, 145.94, 142.36, 141.78, 134.91, 129.57, 128.88, 127.85, 127.52, 122.50, 119.92, 117.10, 113.99, 110.81, 109.79, 51.50, 35.93, 29.79, 27.86, 24.93, 19.90. HRMS (ESI+): *m/z* calcd for $C_{26}H_{25}N_2O_3^+$ $[M]^+$ 413.1860, found 413.1863.

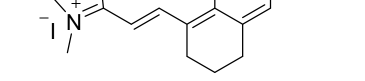

HC-3

HC-3: 39 mg, yield 18%. ^{1}H NMR (500 MHz, $CDCl_3$) δ 8.62 (d, J = 15.6 Hz, 1H), 8.25 (d, J = 8.9 Hz, 1H), 8.16 (s, 1H), 7.52 (s, 4H), 7.34 (d, J = 8.7 Hz, 1H), 7.04 (d, J = 15.2 Hz, 1H), 6.94 (s, 1H), 4.31 (s, 3H), 2.95 (s, 2H), 2.74 (s, 2H), 1.97 (s, 2H), 1.85 (s, 6H). ^{13}C NMR (125 MHz, $CDCl_3$) δ 180.10, 157.12, 156.15, 145.98, 144.19, 142.32, 141.89, 133.21, 129.59, 128.86, 127.37, 125.79, 122.36, 117.04,

116.48, 113.98, 110.19, 51.51, 36.20, 29.63, 27.75, 24.99, 19.92. HRMS (ESI+): *m/z* calcd for $C_{26}H_{25}N_2O_3^+$ $[M]^+$ 413.1860, found 413.1859.

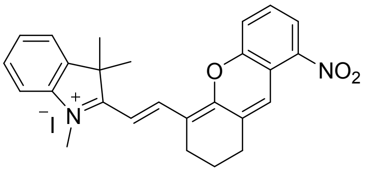

HC-4

HC-4: 45 mg, yield 21%. ^{1}H NMR (500 MHz, $CDCl_3$) δ 8.62 (d, J = 15.5 Hz, 1H), 7.91 (d, J = 8.0 Hz, 1H), 7.69 (s, 1H), 7.54 (tt, J = 13.7, 6.9 Hz, 6H), 6.96 (d, J = 15.4 Hz, 1H), 4.29 (s, 3H), 2.92 (t, J = 6.1 Hz, 2H), 2.81 – 2.72 (m, 2H), 2.03 – 1.93 (m, 2H), 1.84 (s, 6H). ^{13}C NMR (125 MHz, $CDCl_3$) δ 179.96, 156.65, 153.00, 146.00, 144.93, 142.24, 141.94, 135.09, 129.62, 128.77, 123.70, 122.44, 121.59 – 121.42, 121.23, 116.86, 116.44, 113.97, 109.69, 51.49, 36.14, 30.10, 27.73, 24.86, 20.01. HRMS (ESI+): *m/z* calcd for $C_{26}H_{25}N_2O_3^+$ $[M]^+$ 413.1860, found 413.1864.

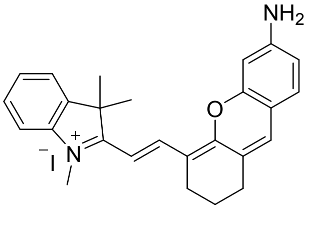

$HC\text{-}NH_2$

$HC\text{-}NH_2$: This compound was synthesized according to the previous literature.[1] ^{1}H NMR (500 MHz, MeOD) δ 8.64 (d, J = 13.2 Hz, 1H), 7.55 (s, 2H), 7.45 (s, 1H), 7.40 – 7.28 (m, 3H), 6.76 (s, 1H), 6.71 (s, 1H), 6.23 (d, J = 13.4 Hz, 1H), 3.69 (s, 3H), 2.75 (dd, J = 39.2, 3.7 Hz, 4H), 1.94 (s, 2H), 1.78 (s, 7H).

Determination of the quantum yield (QY)

The fluorescence quantum yields for the compounds were measured using an Absolute PL Quantum Yield Spectrometer (HAMAMATSU C11347). The PL Quantum Yield (Φ) is expressed as the ratio of the number of photons emitted from molecules (PN_{em}) to those absorbed by the molecules (PN_{abs}).

$\Phi = PN_{em}/PN_{abs}$……………………………………………………………(1)

CLogP (partition coefficient) calculation of fluorophores

The chemical structures of reported probes were collected from the literature. The lipophilic character of the fluorophores was evaluated by determining lipophilicity parameters – partition coefficient (miLogP). CLog P values (miLogP) of fluorophores were calculated using Molinspiration software, which is based on fragmental methods provided by Molinspiration Cheminformatics (available online at www.molinspiration.com). Using the online method, the chemical structures were drawn online, and the properties calculated using Molinspiration property engine v2018.10 to obtain the Clog P values of these molecules.

Enzymatic reduction of HC-NO_2 mediated by nitroreductase (NTR) in the presence of nicotinamide adenine dinucleotide (NADH)

The enzymatic reduction of **HC-NO_2** mediated by nitroreductase (NTR) was performed in phosphate buffer (PB, pH 7.4) containing K_2HPO_4 and KH_2PO_4 (K^+ 100 mM). 200 μL PB containing NTR (0.2 μg/mL), **HC-NO_2** (10 μM), and NADH (0.2 mM) were shaken under constant temperature (37 °C) for 5 min. Then, 100 μL acetonitrile was used to terminate the enzymatic reaction before the removing of denatured protein by centrifugation (20000 *g*). The supernate was subjected to microplate reader for the measurements of fluorescence spectra (λ_{ex} = 670 nm, λ_{em} = 720 nm).

Using the same procedure as above, the fluorescence response of **HC-NO_2** (10 μM) towards different NTR concentrations (0, 0.025, 0.05, 0.075, 0.1, 0.125, 0.15, 0.175, 0.2, 0.3, 0.4, 0.5, 1, 2, 3 μg/mL) were evaluated for the co-incubation (37 °C, 5 min). And, the relationship between the fluorescence intensity and NTR concentrations was calculated. Then, the fluorescence responses of **HC-NO_2** towards NTR for different incubation times (0, 1, 2, 3, 4, 5, 6, 8, 10, 15, 20, 30, 60 min) were evaluated for the co-incubation of **HC-NO_2** (10 μM) and NTR (0.2 μg/mL).The relationship between the fluorescence intensity and incubation time was then calculated.

The selectivity investigation of **HC-NO_2** toward NTR was performed with the co-incubation of **HC-NO_2** and various biological enzymes (CYPs 450) and proteins (BSA, HSA) (1 μg/mL). The interference on the fluorescence intensity of **HC-NO_2** was performed for various oxidative/reductive substances ($NaBH_4$, $Na_2S_2O_3$: 10 μM; Vc, MnO_2, $KMnO_4$, GSH: 1 mM; DTT: 8 mM; H_2O_2: 0.5%; saccharides: 10 mM), ions (10 μM), and amino acids (100 μM).

Investigation about inhibitory effects of compounds on NTR

Pre-incubation was conducted for the PB solution containing NTR, NADH, and compounds to be tested with different concentrations for 3 min at 37 °C. Then, the fluorescent probe **HC-NO_2** (10 μM) was added into the solution to start the enzymatic reaction. The following procedures were the same as mentioned above. Compared with the fluorescence intensity of the control group, the fluorescence intensities of the tested groups were applied to determine the inhibitory rate using GraphPad Prism 6.0.

Fluorescence imaging of bacterial cells by $HC\text{-}NO_2$ using confocal laser scanning microscopy

Bacteria were cultured in LB medium (37 °C, 140 r/min) in order to obtain enough cells with OD_{600} values of 0.7-0.8. The medium was removed by centrifugation (5000 rpm, 10 min). The bacterial cells were washed two times by PB (1 mL) and suspended in PB (1 mL) following pre-incubation for 3 min at 37 °C. Then, **$HC\text{-}NO_2$** was added to the bacterial suspension and incubated at 37 °C for 3 h. For the inhibitory experiments, inhibitors (Alkannin, Plumbagin, Menadione) were added to the bacterial suspensions together with the fluorescent probe. After incubation, the bacterial cells were washed using PB and centrifugation (5000 rpm). The bacterial cells were then resuspended in 30 μL PB for fluorescence imaging experiments.

A bacterial suspension (2 μL) was dropped onto a glass slide, which was then subjected to confocal laser scanning microscopy to obtain the fluorescence images (λ_{ex} = 633 nm, λ_{em} = 690 - 750 nm).

For above mentioned bacterial suspensions, flow cytometric analysis was performed using a Biosciences Accuri C6 flow cytometer (λ_{ex} = 640 nm, λ_{em} > 670 nm).

Fluorescence imaging of bacterial colonies by $HC\text{-}NO_2$ on agar plates

Various bacteria were cultured in a liquid medium to afford a stock bacterial suspension (OD_{600} 0.8). After dilution ($10^5\times$), the bacterial strains were coated on to agar plates as culture medium followed by cultivation at 37 °C for 24 h. For anaerobic bacteria, the cultivation was conducted in an anaerobic box at 37 °C for 48 h. The bacterial colonies were observed by naked eye. **$HC\text{-}NO_2$** solution was dropped onto the bacterial colonies carefully followed by 1h incubation at 37 °C. For inhibitory experiments (menadione, 50 μM), the inhibitor was added at the same time. After the co-incubation, the agar plates were then imaged using a fluorescence scanner (λ_{ex} = 635 nm, λ_{em} = 720 ± 20 nm).

Native-PAGE electrophoresis for bacterial lysates and NTR proteins

Three anaerobic bacteria (*B. fragilis*, *B. thetaiotaomicron*, and *B. bifidum*), and seven aerobic bacteria (*Pseudomonas aeruginosa*, *Escherichia coli 0377*, *Bacillus cereus*, *Staphylococcus hominis*, *Enterococcus faecalis*, *Escherichia coli 3079*, *Klebsiella*

pneumoniae) were cultured at 37 °C for the preparation of bacterial lysates. For various bacterial species, enough cells were obtained in the liquid medium. Then, the cells were ruptured using ultrasonic disruption in an ice bath. After centrifugation at 20000 g for 10 min at 4 °C, the supernatant bacterial lysates were obtained, and the concentration of total proteins was determined using the BCA method.

The native gel was prepared containing 10% polyacrylamide separation gel and 4% concentrated gel. After solidification at room temperature, bacterial lysates containing equal amounts of protein or the recombinant NTR enzyme were loaded into the gel, respectively. The loaded volume of marker sample is 3 μL. The electrophoresis of native PAGE-gel was carried out under constant pressure (100 V for 0.5 h and 150 V for 1 h) with ice-water bath until the marker strips are separated and the bromophenol blue was electrophoresed to the bottom of the gel.

When the electrophoresis of gel was finished, the PAGE was subjected to a **HC-NO$_2$** solution (10 μM) in the presence of NADH (0.2 mM) and incubated at 37 °C for 30 min. After washing using PB solution, the gel was scanned using a fluorescence scanner and fluorescence images were obtained (λ_{ex} = 635 nm, λ_{em} = 720 ± 20 nm).

For the fluorescence bands, the gel bands were cut and stored at -80 °C. Then, the gel bands were subjected to LC-MS/MS for protein determination. Combined with the UNIPROT database, the measured data were analyzed and the predicted NTRs were determined for these gel bands.

Furthermore, the whole genome of four bacteria (*B. thetaiotaomicron*; *B. fragilis*; *E. coli 0377*; *B. cereus*) were sequenced and assembled. Then on the basis of reported NTR from various species in the UNIPROT database, the NTRs for these bacteria were annotated according to their whole genome.

Table S1. Calculated log P and excitation/emission wavelength of reported fluorophores.

No.	Fluorophores	ClogP	$\lambda_{ex}/\lambda_{em}$ (nm)
**1**	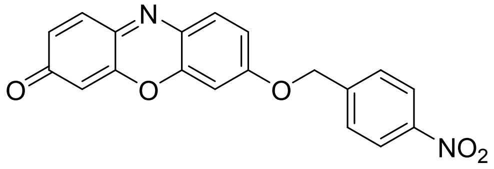	4.23	564/586
**2**	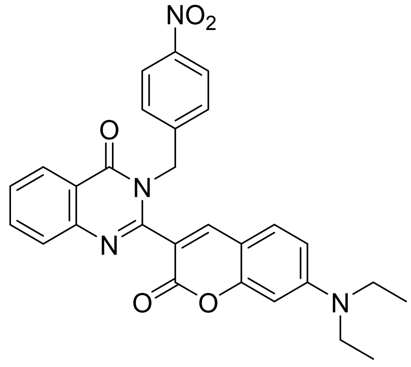	5.56	460/505
**3**	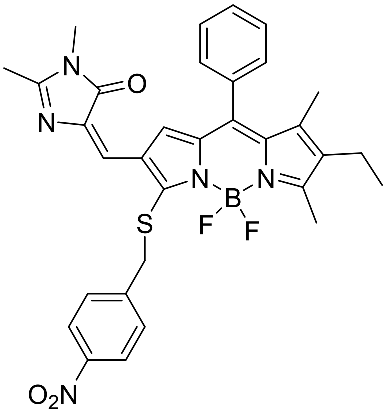	-0.49	675/725
**4**	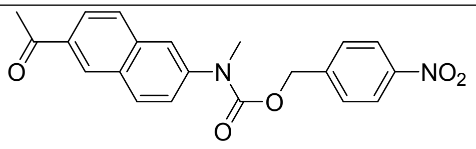	4.5	410/500
**5**	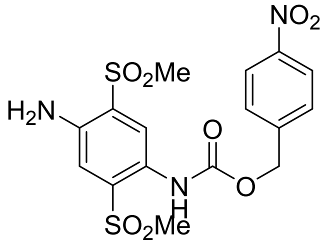	1.16	375/520

**6**	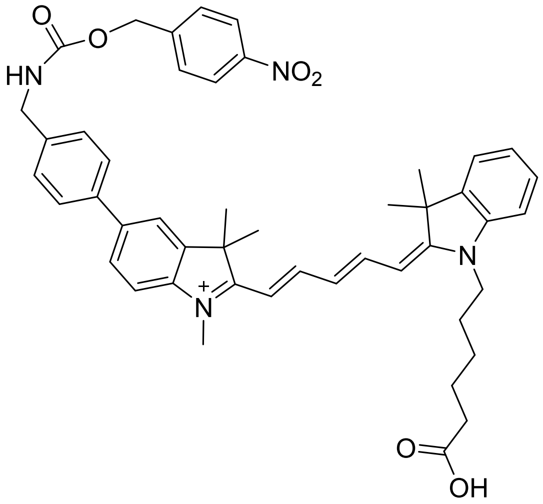	6.82	620/657
**7**	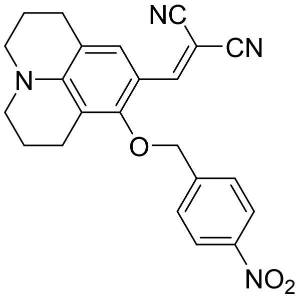	4.61	330/492
**8**	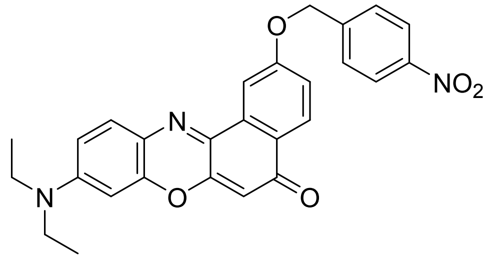	6.17	585/655
**9**	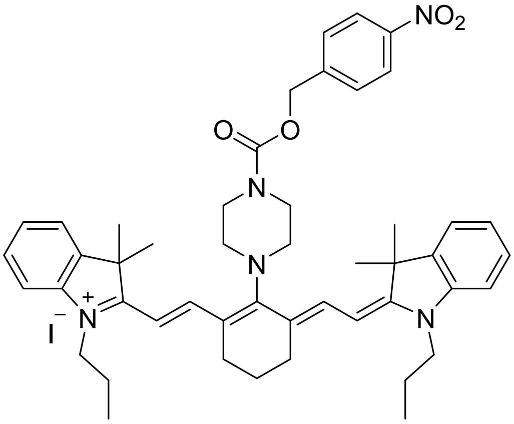	7.78	710/785

**10**	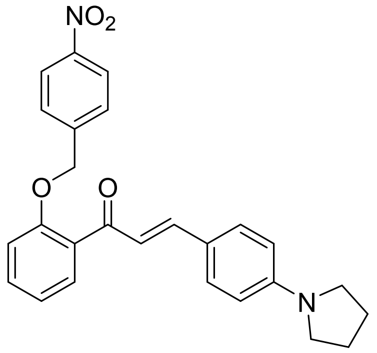	5.88	467/526
**11**	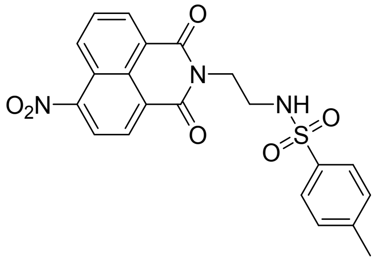	3.11	440/543
**12**	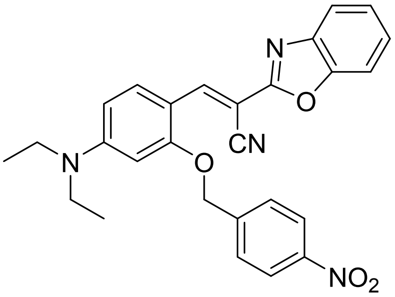	6.03	455/530
**13**	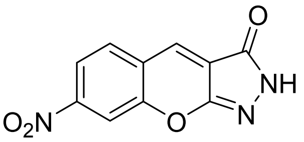	1.74	375/505
**14**	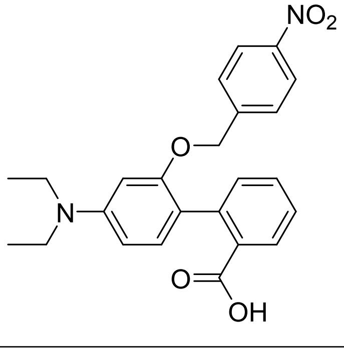	5.65	380/530
**15**	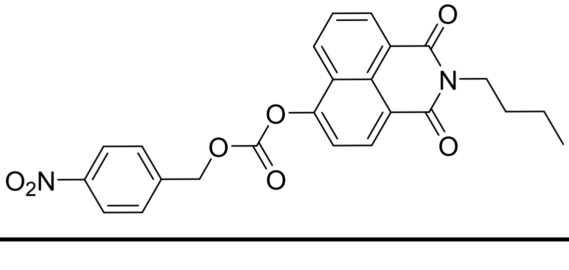	4.91	450/550

**16**	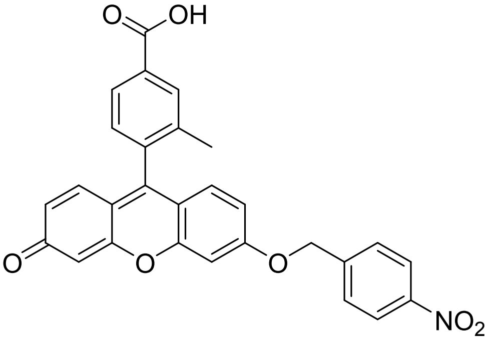	6.77	479/515
**17**	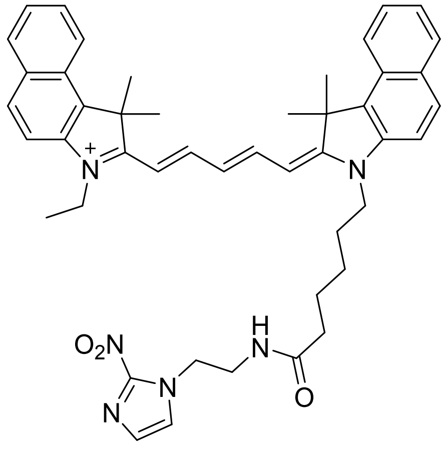	6.61	658/699
**18**	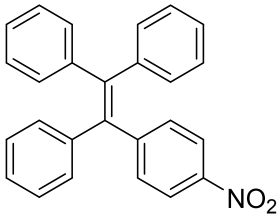	6.65	405/525
**19**	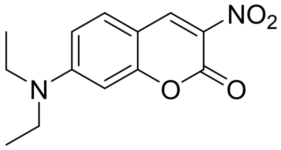	2.73	380/511
**20**	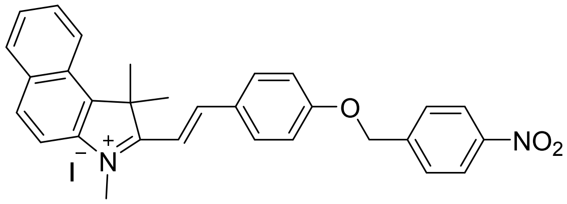	4.05	523/570
**21**	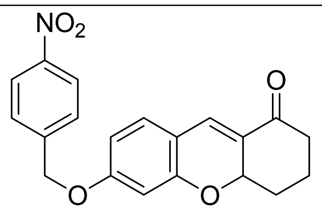	3.94	450/511

**22**	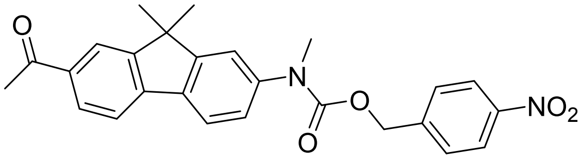	5.62	365/565
**23**	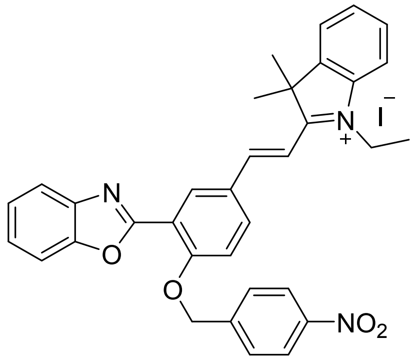	4.91	530/580
**24**	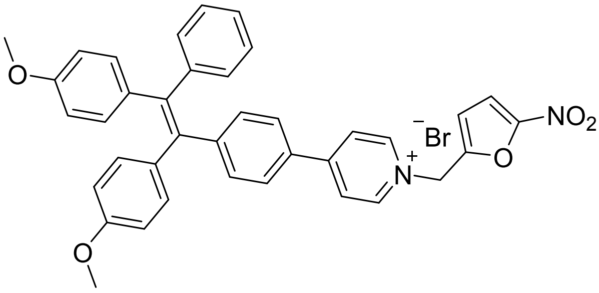	3.08	450/525
**25**	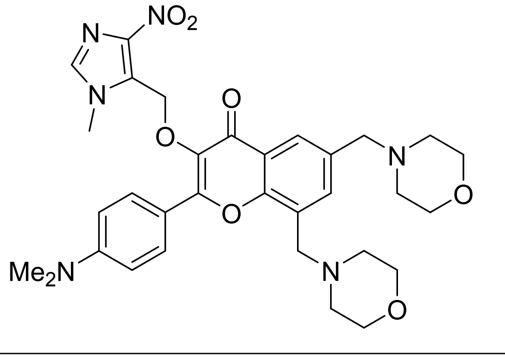	2.16	412/560
**26**	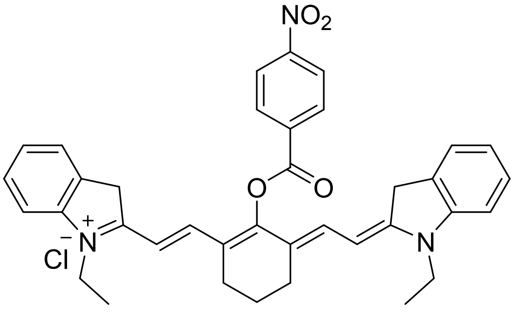	5.46	730/782
**27**	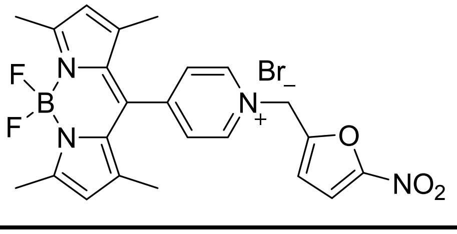	-5.56	470/520

**28**	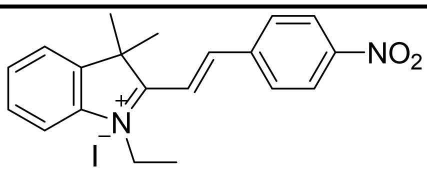	1.62	490/556
**29**	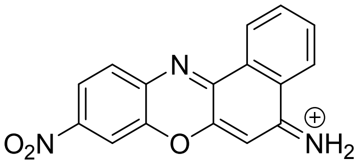	1.14	585/625
**30**	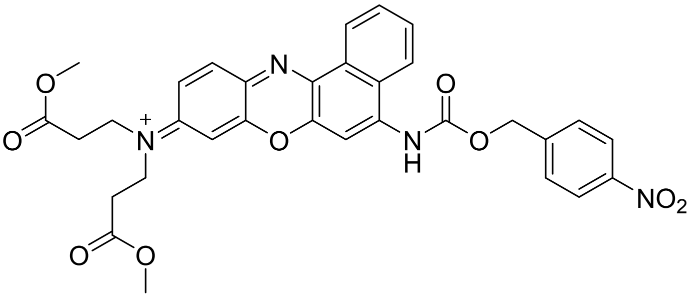	1.37	580/658
**31**	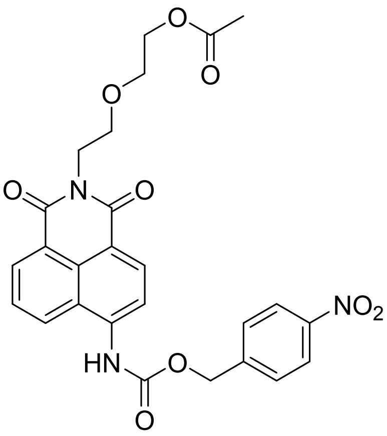	3.14	410/550
**32**	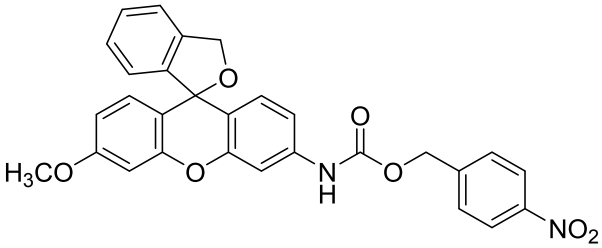	6.09	467/519
**33**	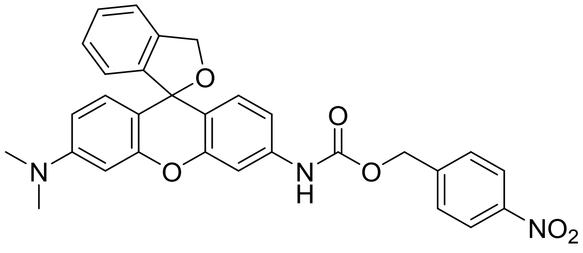	6.88	504/556

**34**	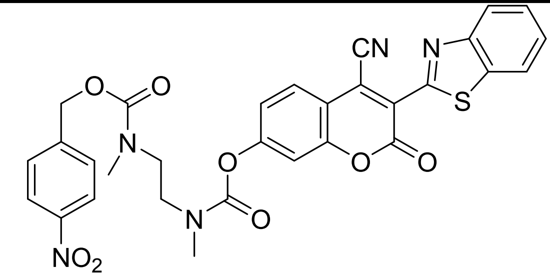	5.01	500/595
**35**	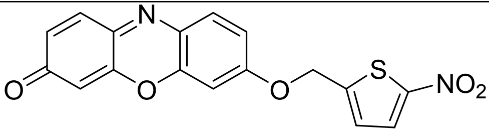	4.25	550/585
**36**	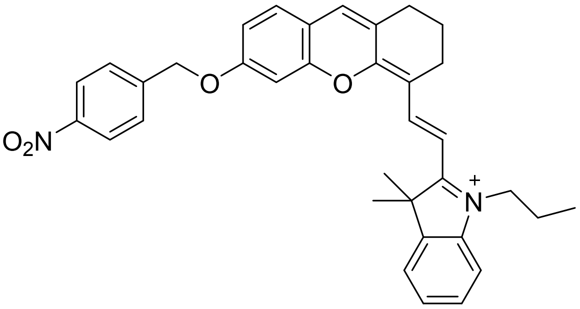	5.15	670/705
**37**	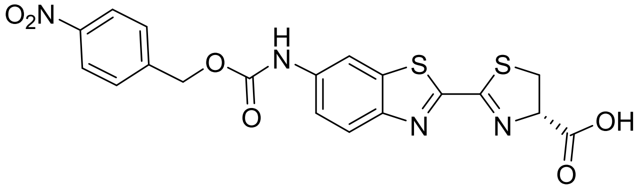	1.4	---/525
**38**	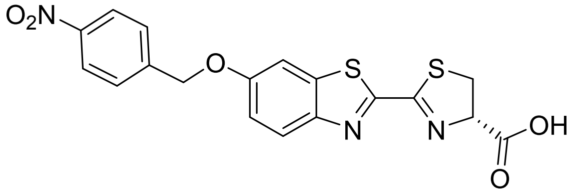	1.75	---/525
**39**	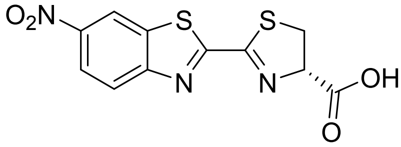	0.09	---/525
**40**	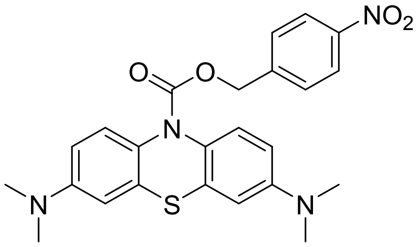	5.64	580/680
**41**	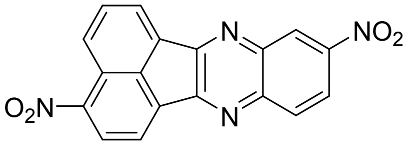	4.77	361/526

**42**	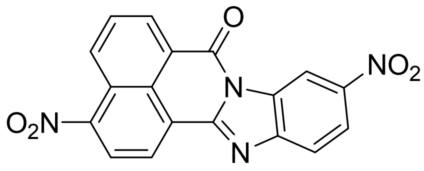	4.06	465/520
**43**	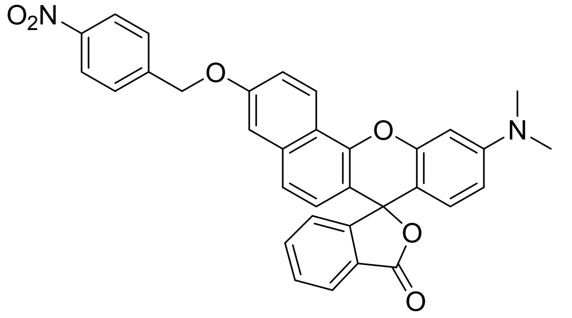	7.52	534/583
**44**	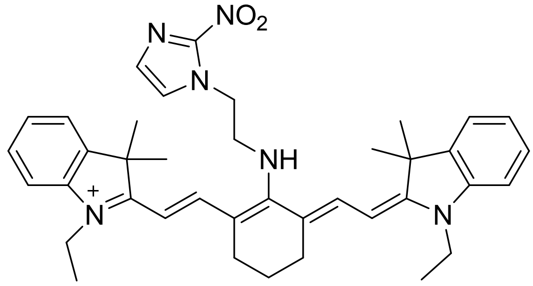	5.2	695/750
**45**	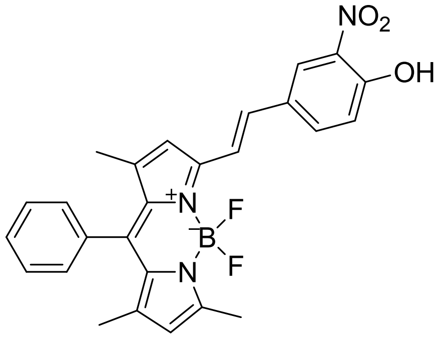	-0.18	543/585
**46**	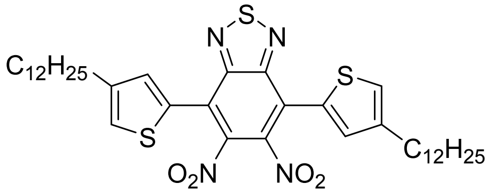	10.26	480/600
**47**	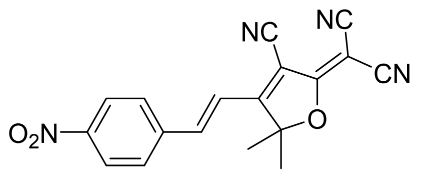	3.78	500/643

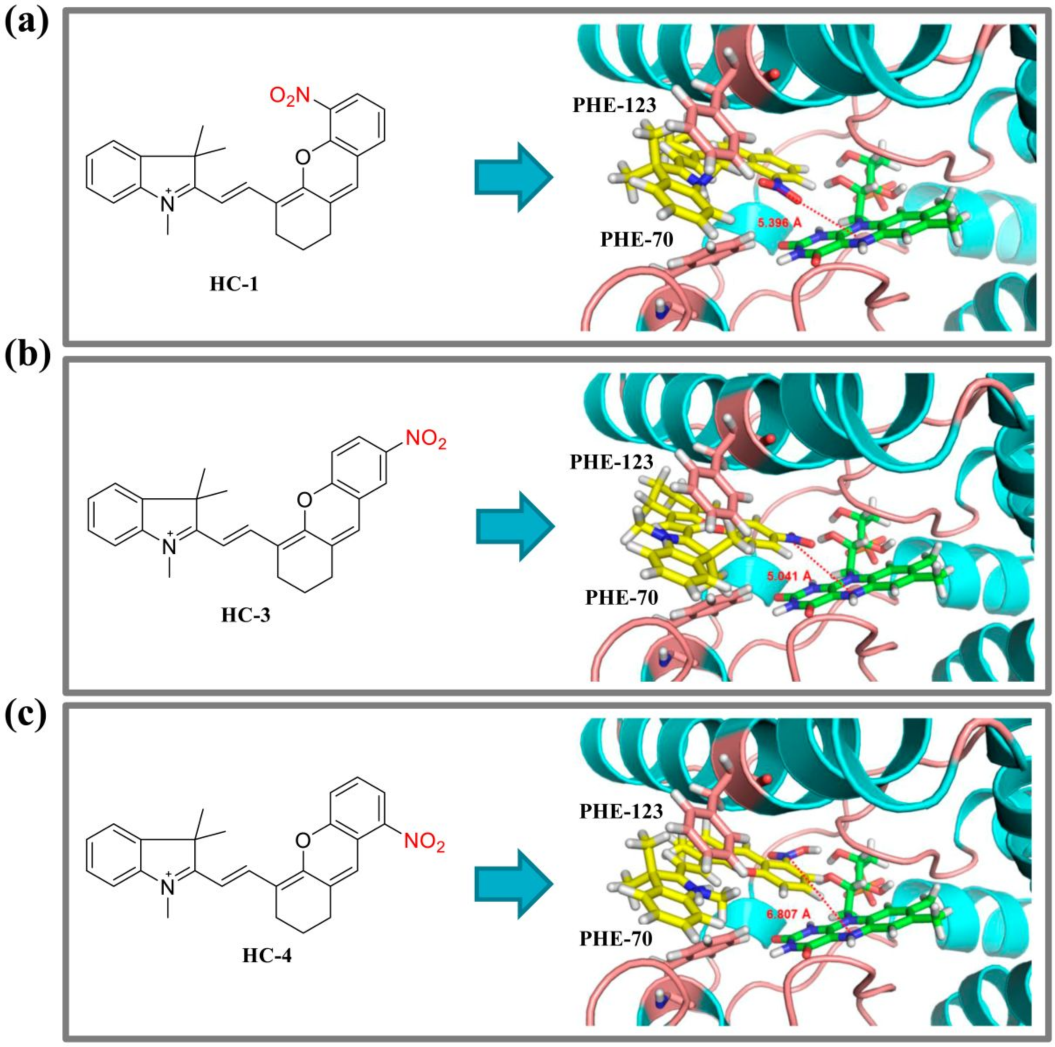

Figure S1. Chemical structures of **HC-1** (a), **HC**-3 (b), **HC-4** (c) and *in silico* docking analysis between these molecules and NTR protein.

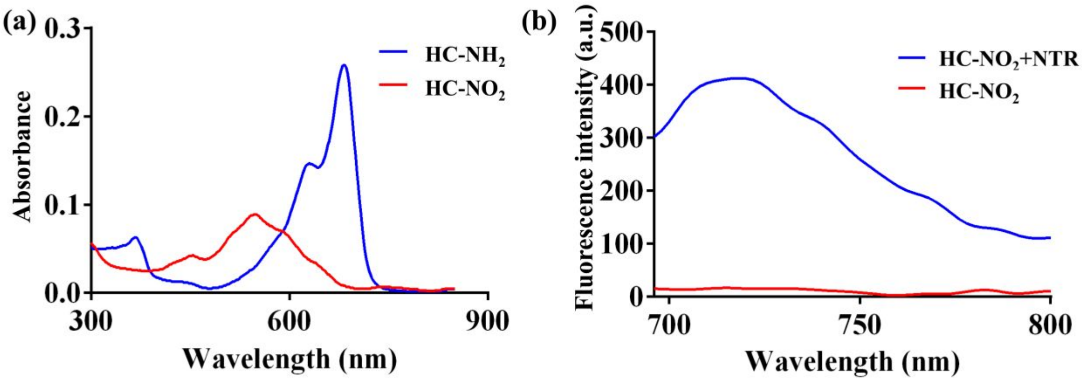

Figure S2. (a) The absorption spectra of **HC-NO_2** and **HC-NH_2**. (b) The fluorescence spectra of **HC-NO_2** and enzymatic reduction of **HC-NO_2** mediated by NTR (Excitation = 670 nm).

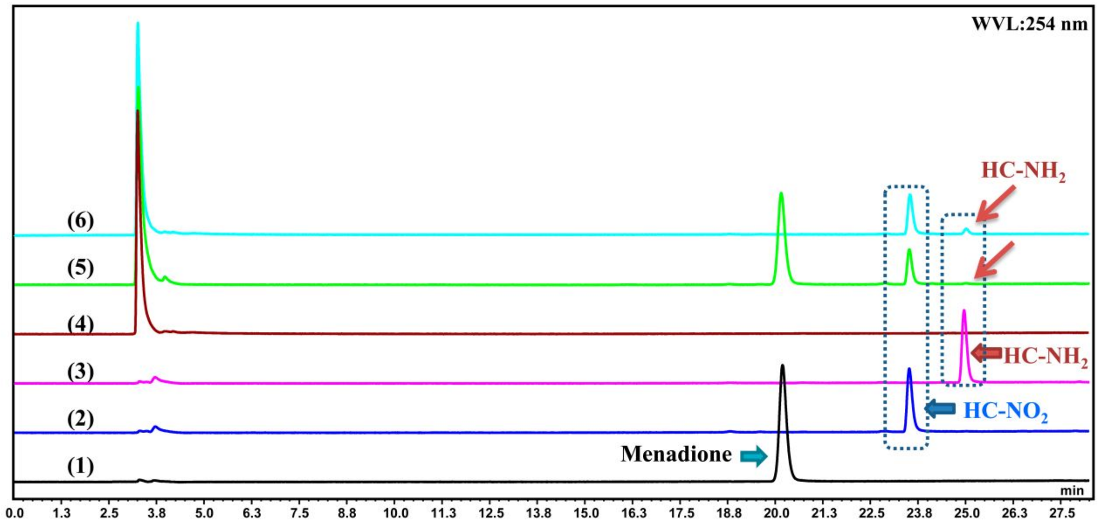

Figure S3. HPLC chromatograms for the enzymatic reaction of **HC-NO$_2$** for the nitroreductase co-incubation culture. (1) Standard reference of menadione; (2) Standard reference of **HC-NO$_2$**; (3) Standard reference of **HC-NH$_2$**; (4) Blank culture of NTR; (5) Co-incubation of **HC-NO$_2$** and NTR together with NADH in the presence of menadione; (6) Co-incubation of **HC-NO$_2$** and NTR together with NADH.

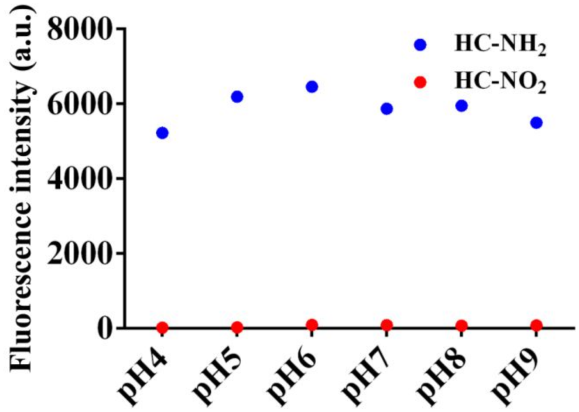

Figure S4. The effect of pH on the fluorescence intensity of **HC-NO$_2$** and **HC-NH$_2$** (10 μM) $\lambda_{ex.}$ 670 nm, $\lambda_{em.}$ 720 nm.

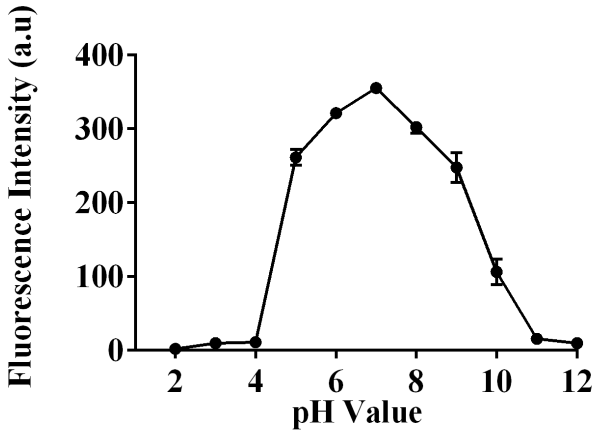

Figure S5. The fluorescence intensities for the co-incubations of **$HC\text{-}NO_2$** and NTR in phosphate solution at different pH values ($\lambda_{ex.}$ 670 nm, $\lambda_{em.}$ 720 nm).

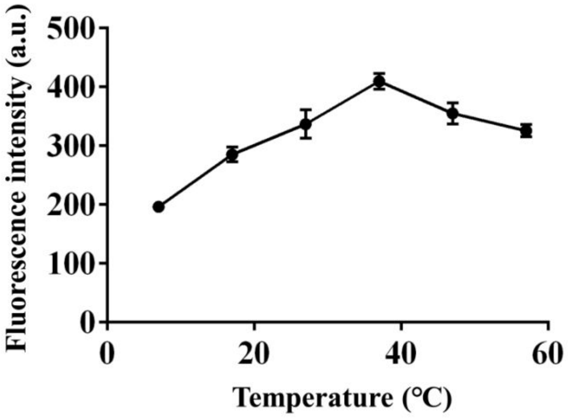

Figure S6. The fluorescence intensities for the co-incubations of **$HC\text{-}NO_2$** and NTR in phosphate solution at different temperature ($\lambda_{ex.}$ 670 nm, $\lambda_{em.}$ 720 nm).

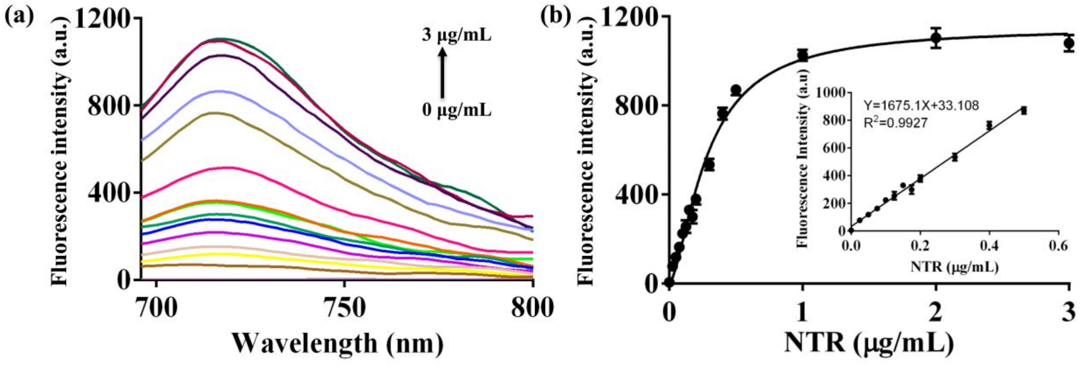

Figure S7. (a) The fluorescence responses of **$HC\text{-}NO_2$** towards different NTR concentrations (0, 0.025, 0.05, 0.075, 0.1, 0.125, 0.15, 0.175, 0.2, 0.3, 0.4, 0.5, 1, 2, 3 μg/mL). (b) The relationship between the fluorescence intensity and NTR concentrations.

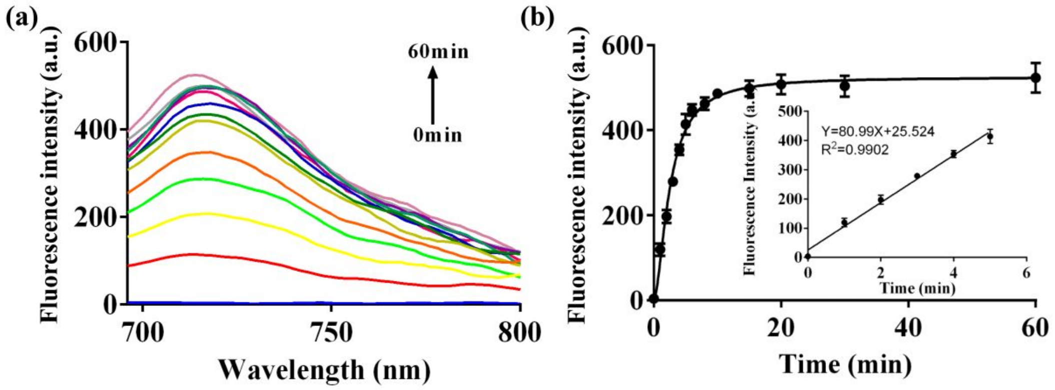

Figure S8. (a) The fluorescence responses of **HC-NO$_2$** towards NTR for different incubation times (0, 1, 2, 3, 4, 5, 6, 8, 10, 15, 20, 30, 60 min). (b) The relationship between the fluorescence intensity and incubation time.

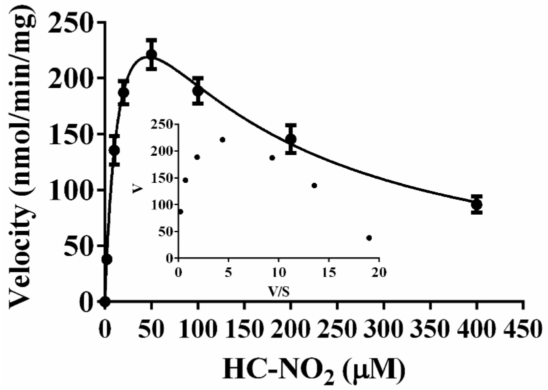

Figure S9. Kinetics about the enzymatic reduction of **HC-NO$_2$** mediated by NTR.

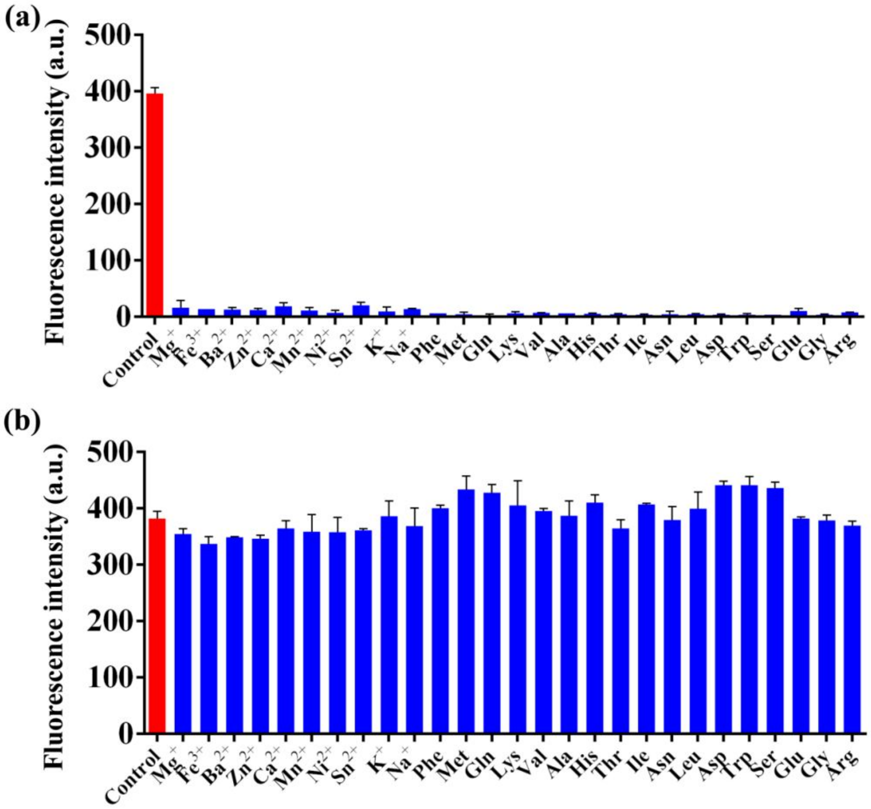

Figure S10. (a) The fluorescence response of **HC-NO$_2$** towards various species (ions and amino acids). (b) The fluorescence intensity for the co-incubation of **HC-NO$_2$** and NTR in the presence of various species (ions and amino acids). Control group: co-incubation of **HC-NO$_2$** and NTR.

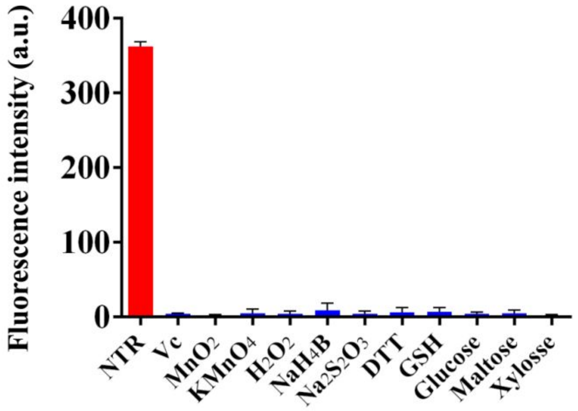

Figure S11. The fluorescence response of **HC-NO$_2$** toward various redox agents. Control group: co-incubation of **HC-NO$_2$** and NTR.

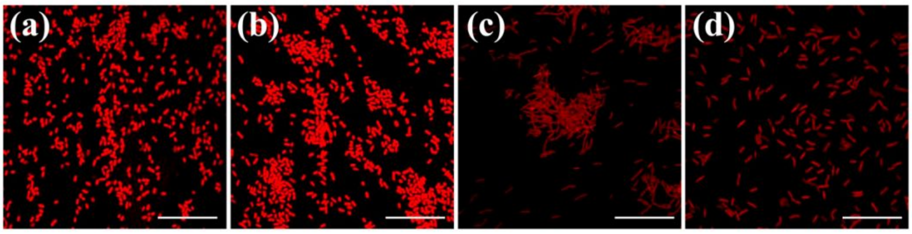

Figure S12. Confocal laser scanning microscopic images of various general bacterial cells stained using **HC-NO$_2$**. (a) *Escherichia coli* 0377, (b) *Streptococcus lactis*, (c) *Streptococcus haemolyticus*, (d) *Lactobacillus salivarius*. $\lambda_{ex.}$ 633 nm, $\lambda_{em.}$ 690-750 nm, Scale bar 25 μm.

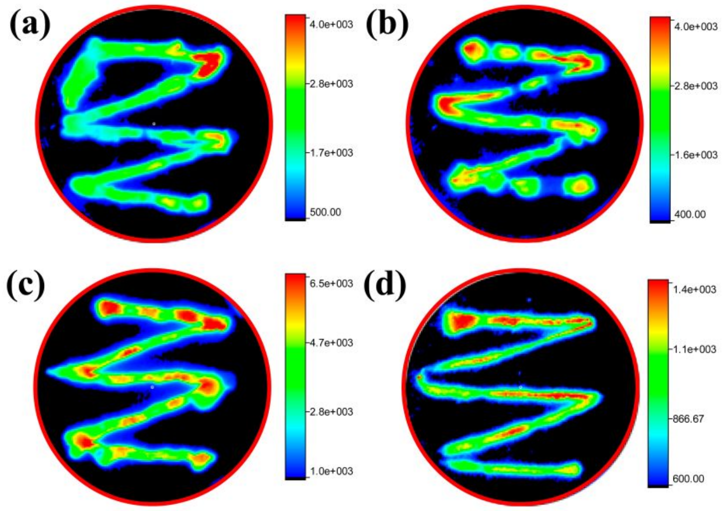

Figure S13. Fluorescence images of various general bacterial colonies on agar plates stained using **HC-NO$_2$**. (a) *Escherichia coli* 0377, (b) *Streptococcus lactis*, (c) *Streptococcus haemolyticus*, (d) *Lactobacillus salivarius*.

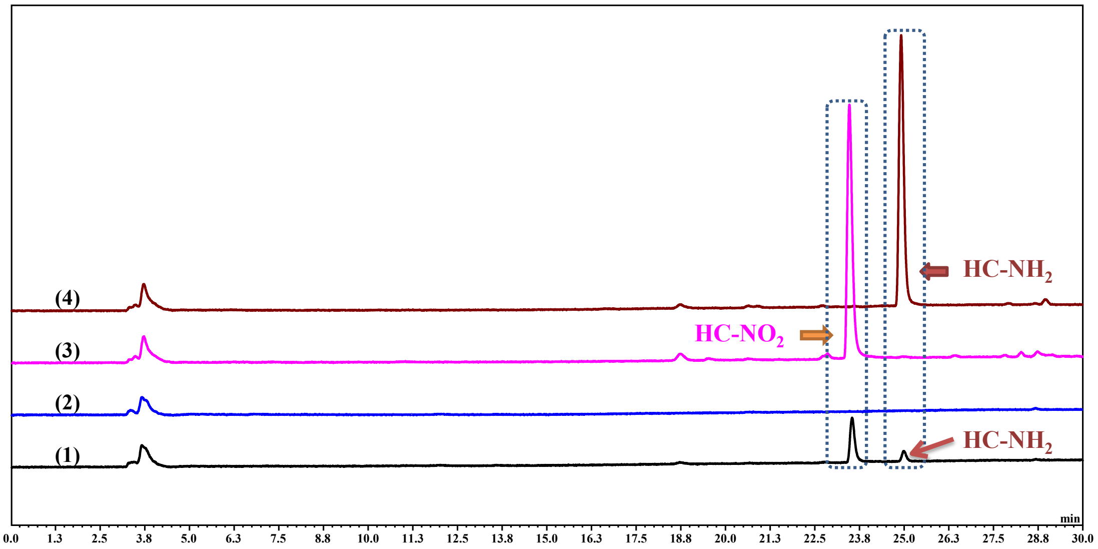

Figure S14. HPLC chromatograms of **HC-NO$_2$** metabolites from *Escherichia coli* 4164 culture. (1) *Escherichia coli* 4164 culture in the presence of **HC-NO$_2$**; (2) Blank culture of *Escherichia coli* 4164; (3) Standard reference of **HC-NO$_2$**; (4) Standard reference **HC-NH$_2$**.

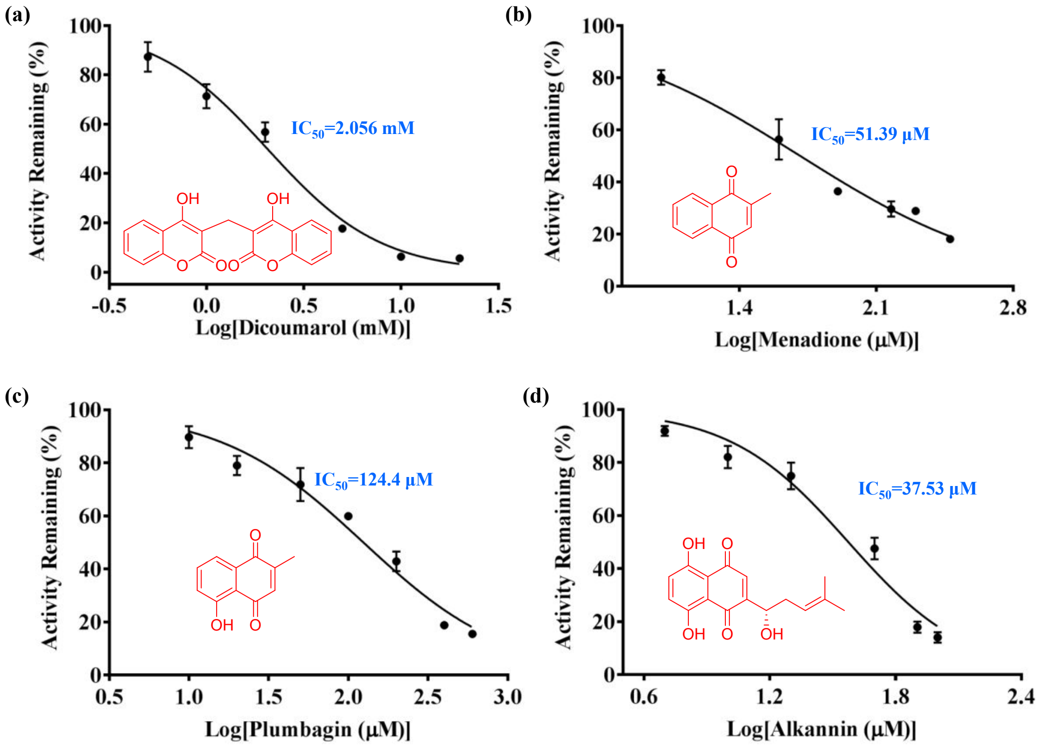

Figure S15. The inhibitory effects of four inhibitors against NTR. (a) Dicoumarol, (b) Menadione, (c) Plumbagin, (d) Alkannin.

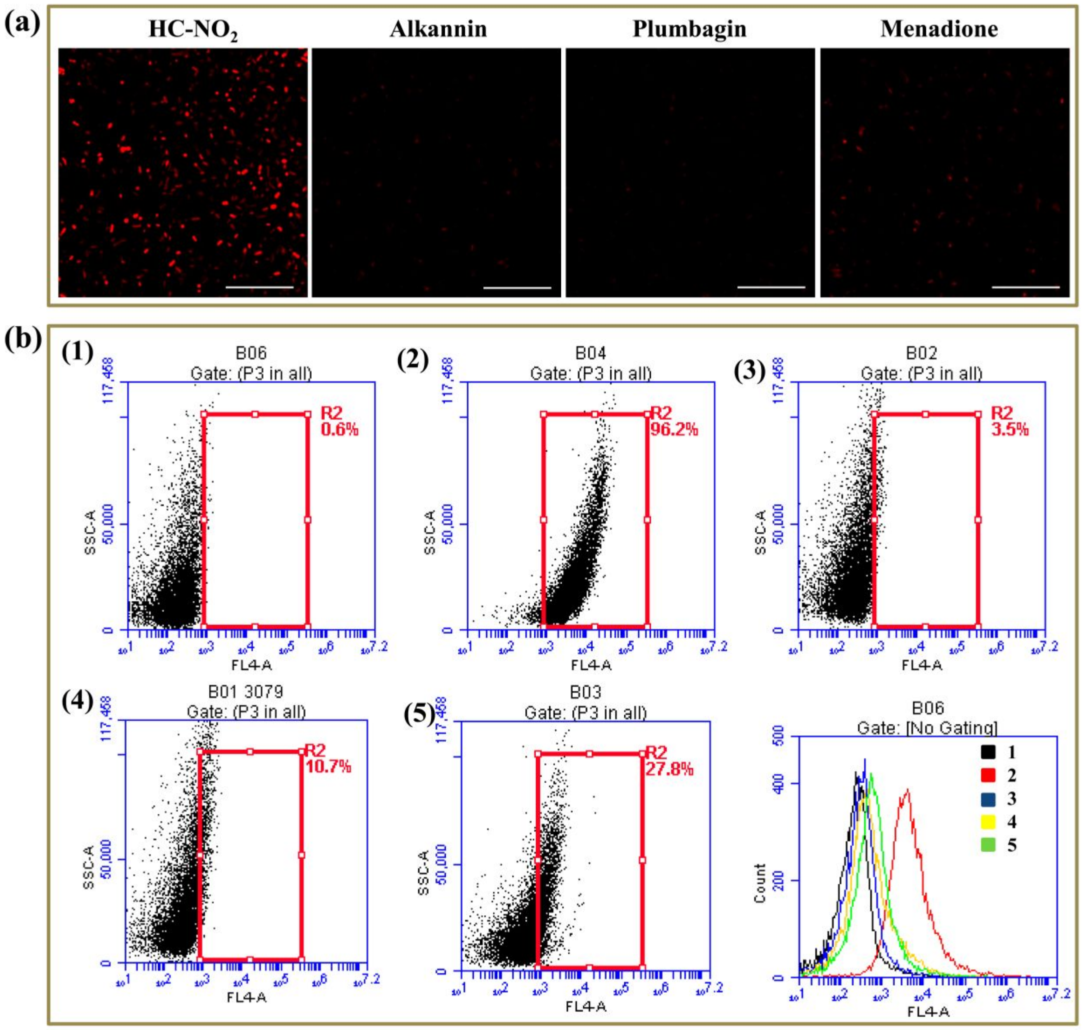

Figure S16. (a) Confocal laser scanning microscopic images and (b) flow cytometric analysis of *E. faecalis* stained by **HC-NO$_2$** in the presence of inhibitors. Flow cytometric graph: blank group (1), control group (2), alkannin (3), plumbagin (4), menadione (5). Scale bar 25 μm.

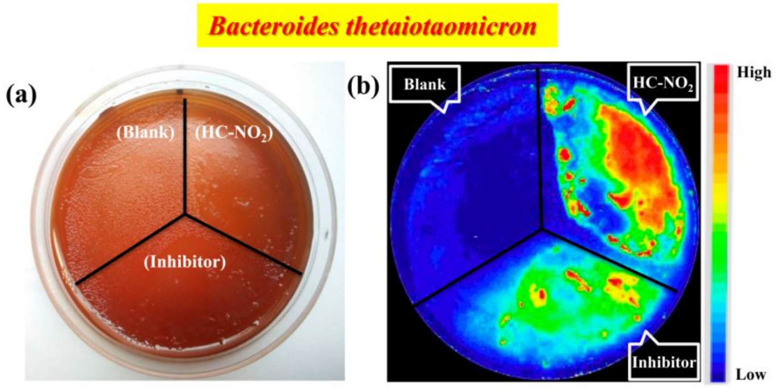

Figure S17. (a) Bright image and (b) fluorescence image of *Bacteroides thetaiotaomicron* on an agar plate in the presence of **HC-NO$_2$** and inhibitor (menadione).

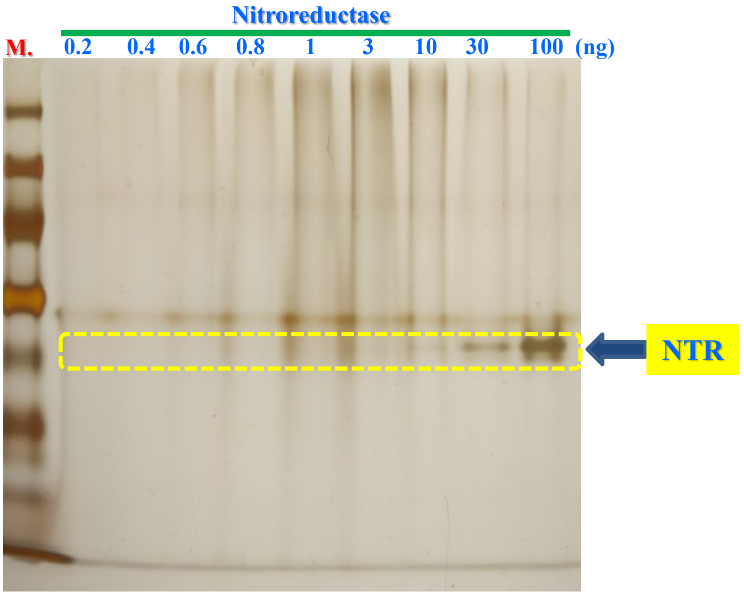

Figure S18. Sensitivity investigation for NTR assay on native PAGE-gel stained using silver.

Table S2. Inhibitory effect of metronidazole on various bacteria.

No.	Bacterial name	MIC (μg/mL)
1	*Bacteroides fragilis*	0.5
2	*Bacteroides thetaiotaomicron*	1
3	*Bifidobacterium bifidum*	1
4	*Pseudomonas aeruginosa*	>64
5	*Escherichia coli 0377*	>64
6	*Bacillus cereus*	>64
7	*Staphylococcus hominis*	>64
8	*enterococcus faecalis*	>64
9	*Escherichia coli*	>64
10	*Klebsiella pneumoniae*	>64

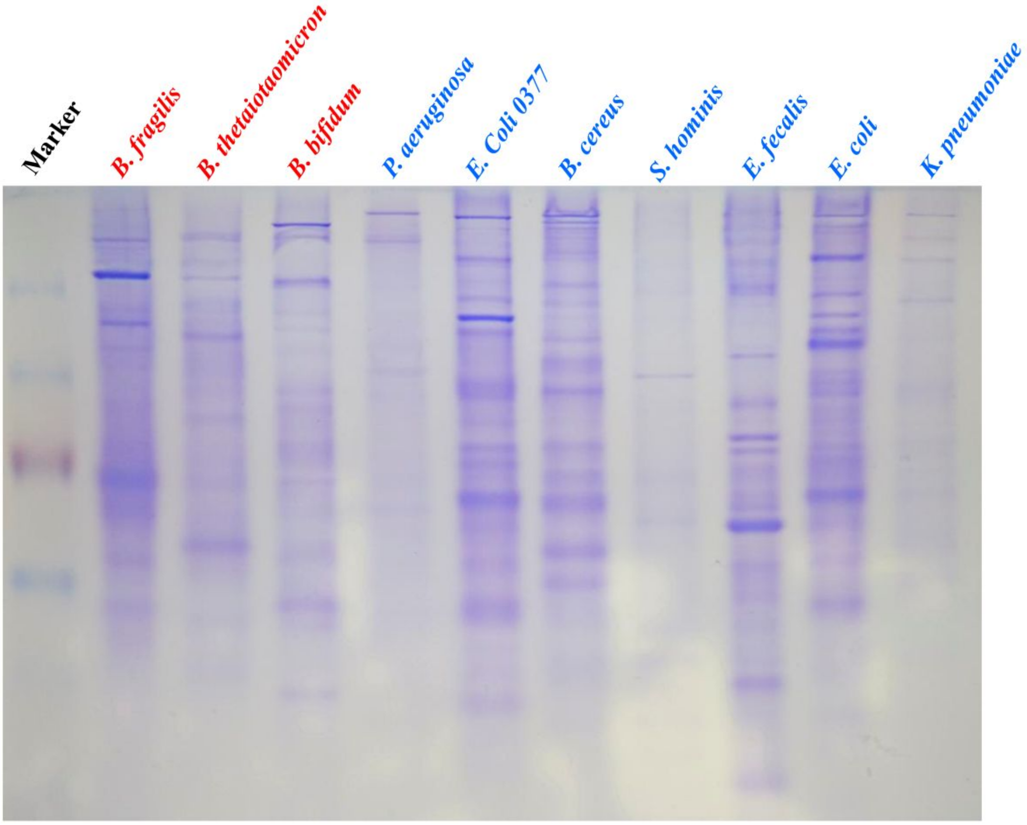

Figure S19. Total protein profiling of various bacteria species on native gel stained using Coomassie brilliant blue.

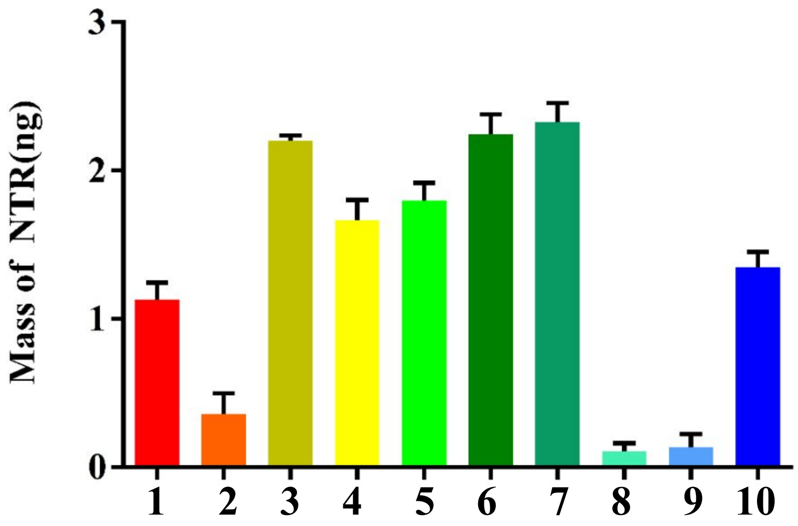

Figure S20. NTR expression of various bacteria species determined by native PAGE-gel using fluorescent probe **HC-NO$_2$** for staining. (1) *B. fragilis*, (2) *B. thetaiotaomicron*, (3) *B. bifidum*, (4) *P. aeruginosa*, (5) *E. coli 0377*, (6) *B. cereus*, (7) *S. hominis*, (8) *E. faecalis*, (9) *E. coli*, (10) *K. pneumoniae*.

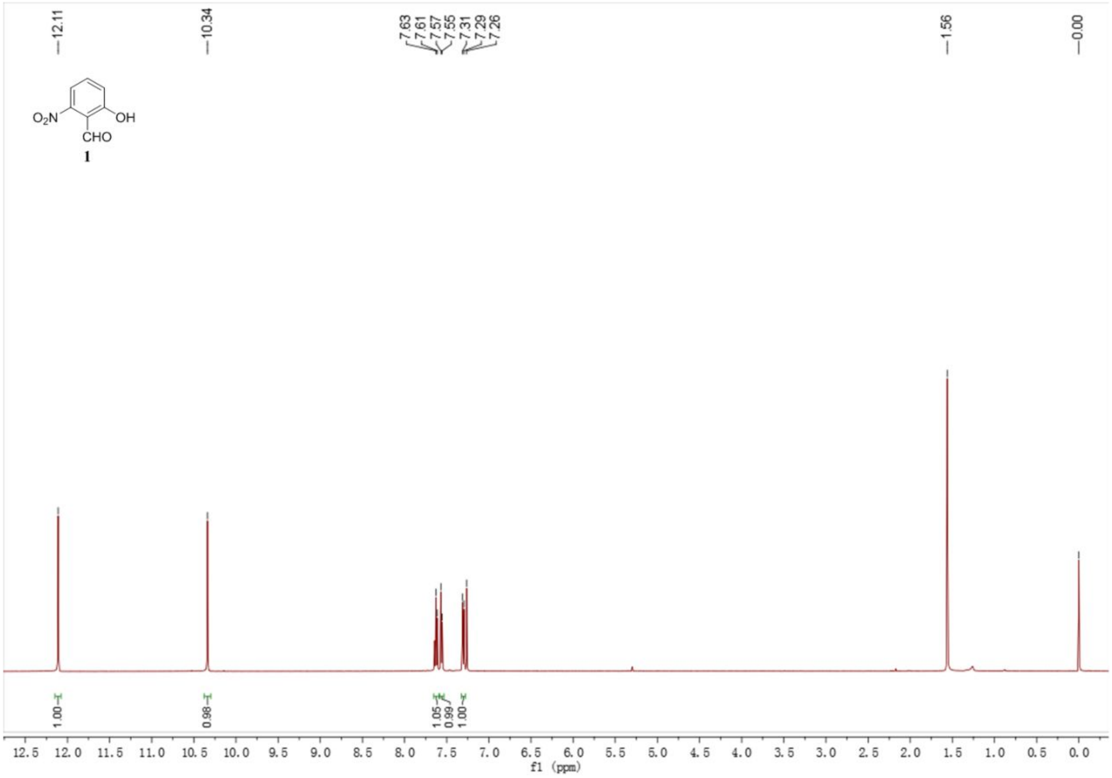

Figure S21. ^{1}H NMR spectrum of compound **1** ($CDCl_3$).

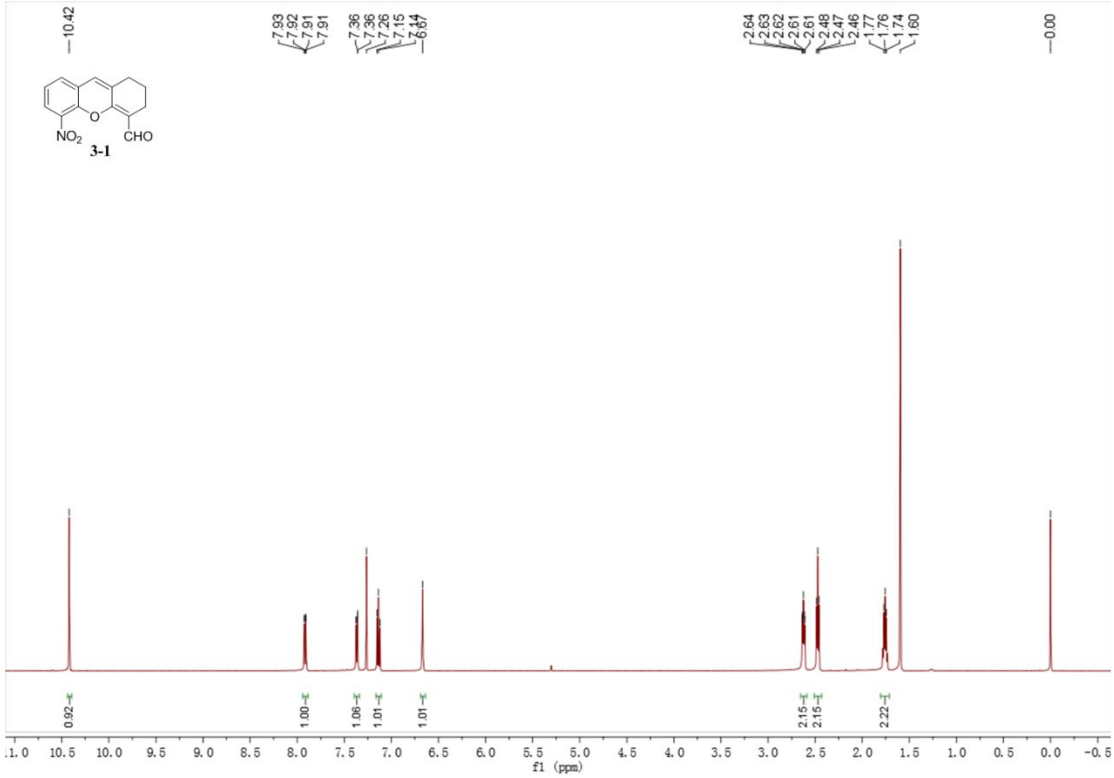

Figure S22. ^{1}H NMR spectrum of **3-1** ($CDCl_3$).

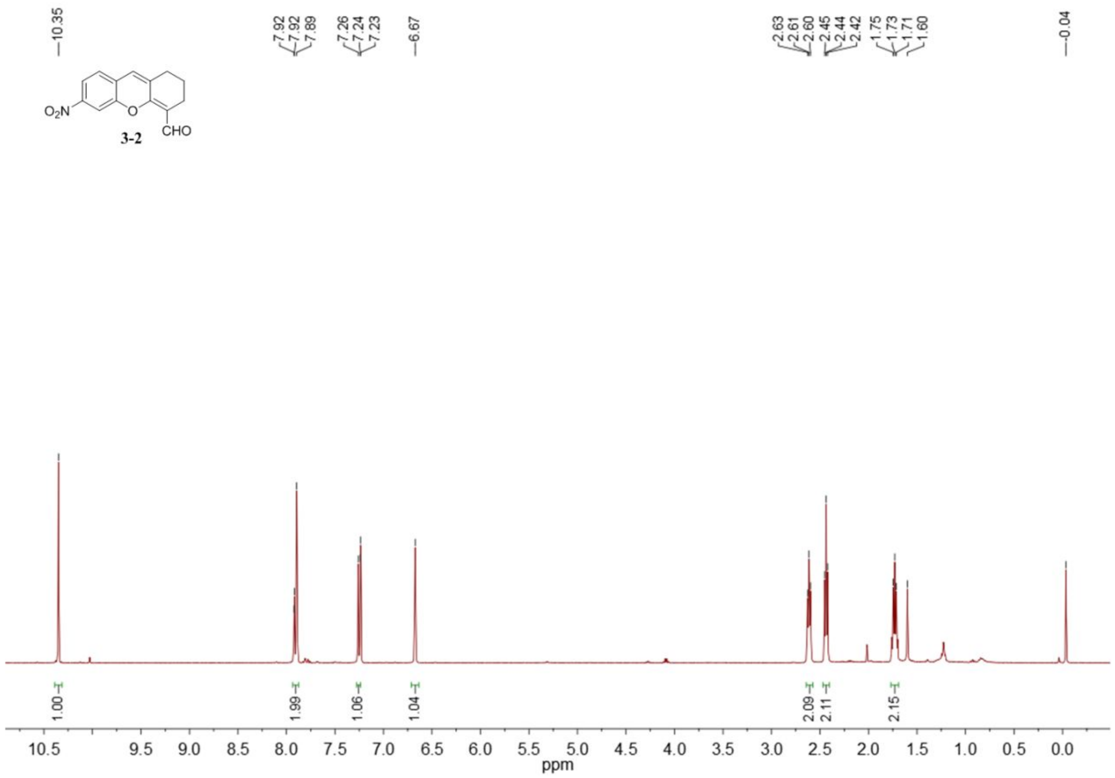

Figure S23. ^{1}H NMR spectrum of **3-2** ($CDCl_3$).

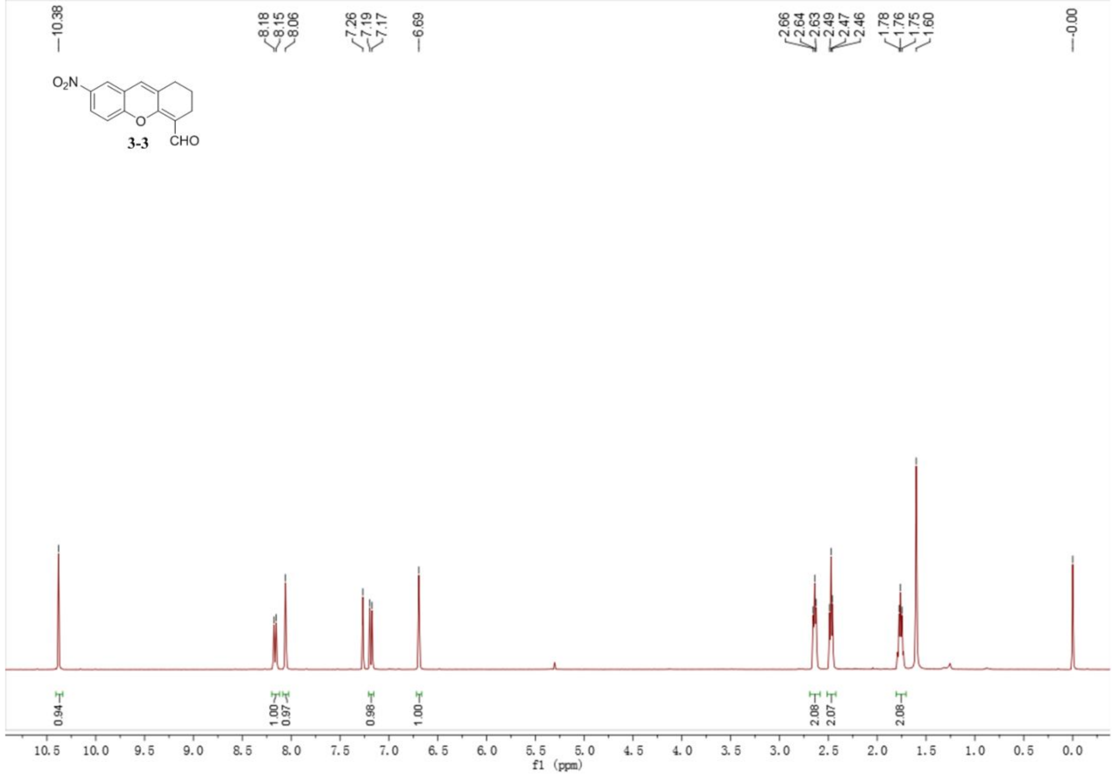

Figure S24. ^{1}H NMR spectrum of **3-3** ($CDCl_3$).

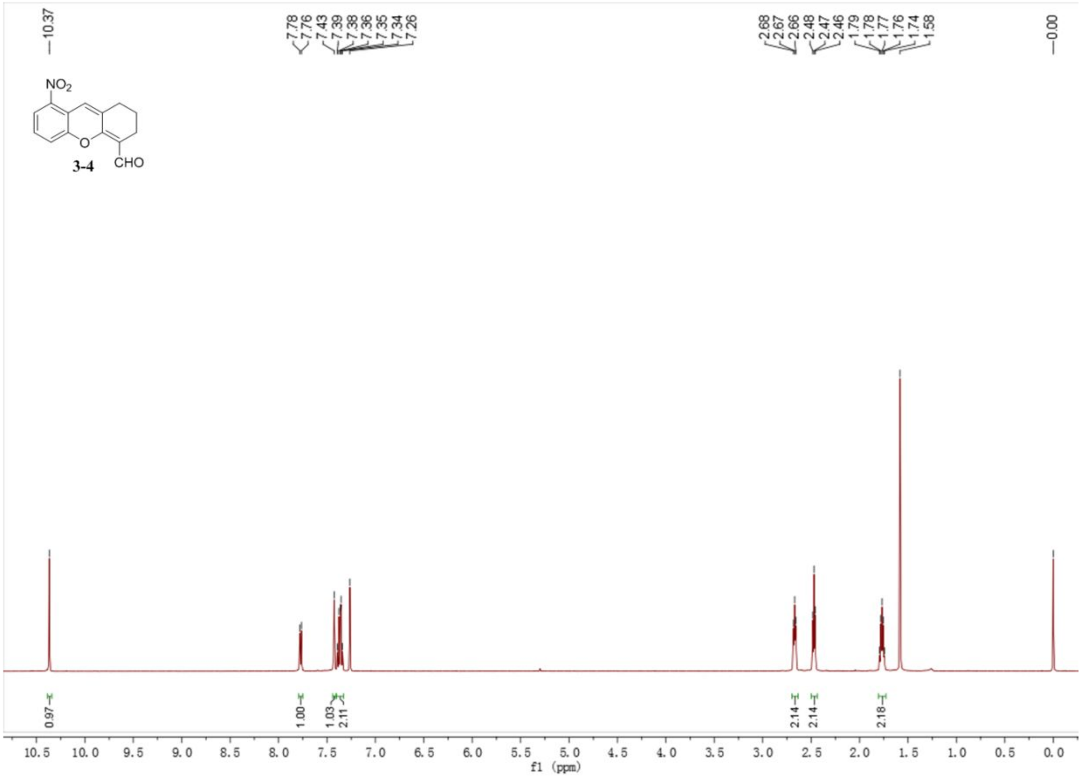

Figure S25. ^{1}H NMR spectrum of **3-4** ($CDCl_3$).

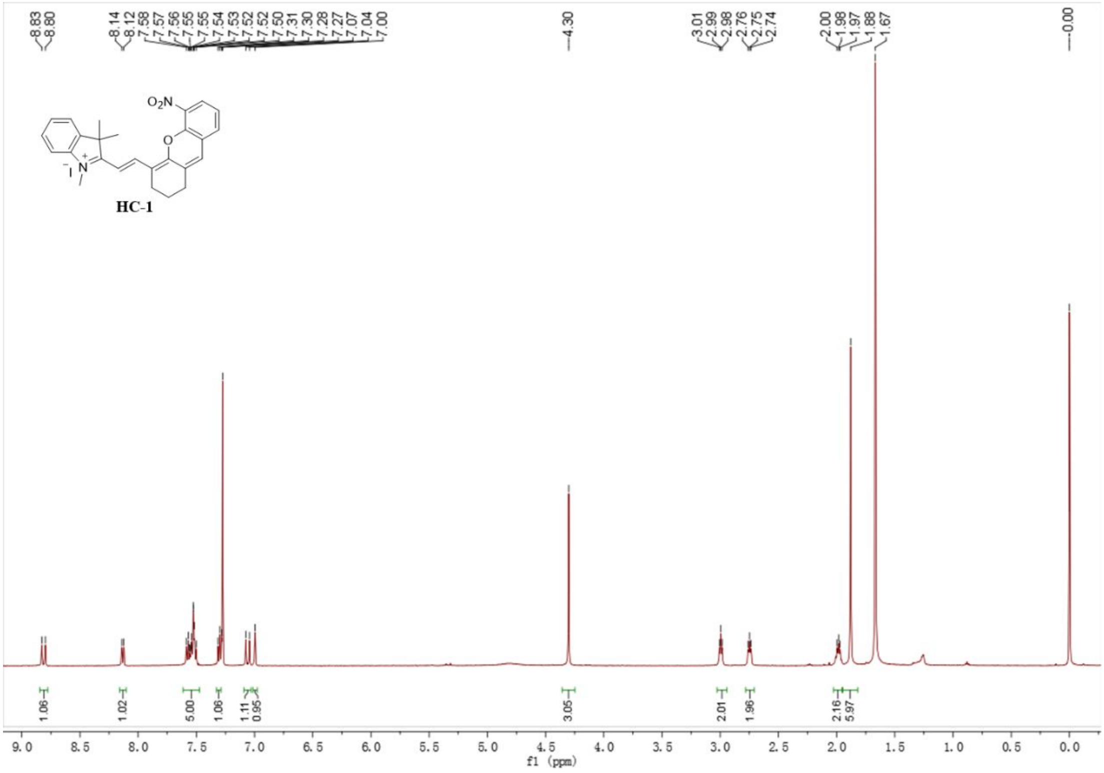

Figure S26. ^{1}H NMR spectrum of **HC-1** ($CDCl_3$).

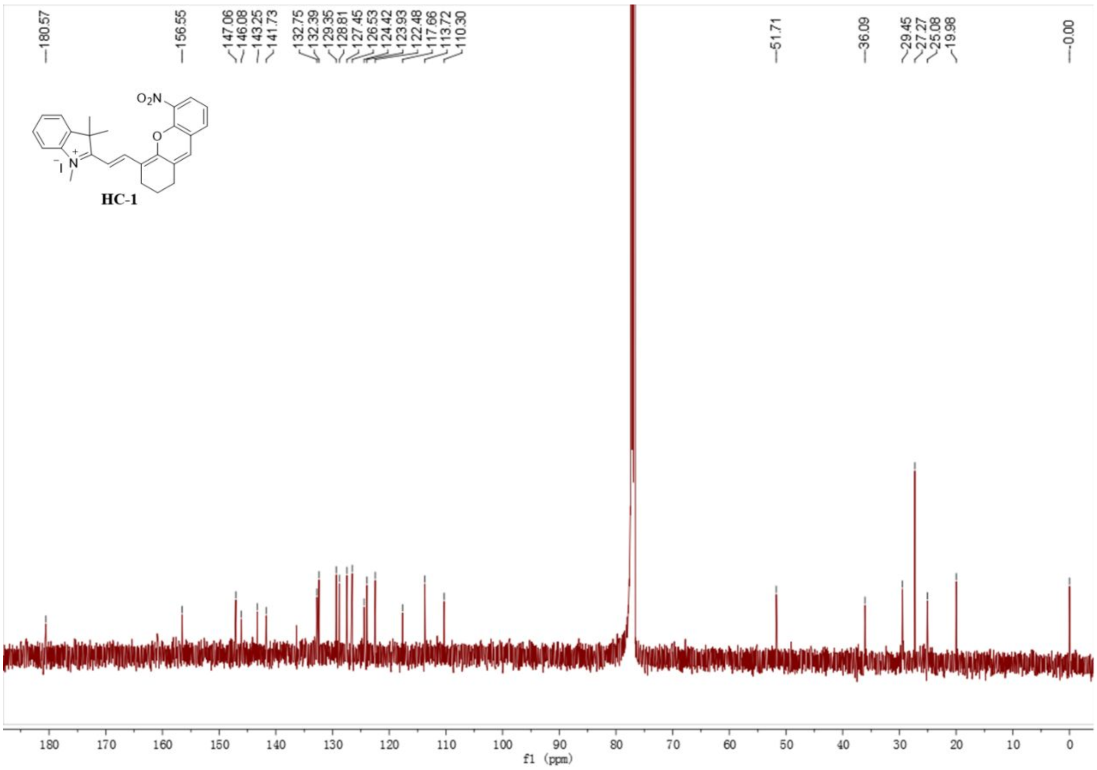

Figure S27. ^{13}C NMR spectrum of **HC-1** ($CDCl_3$).

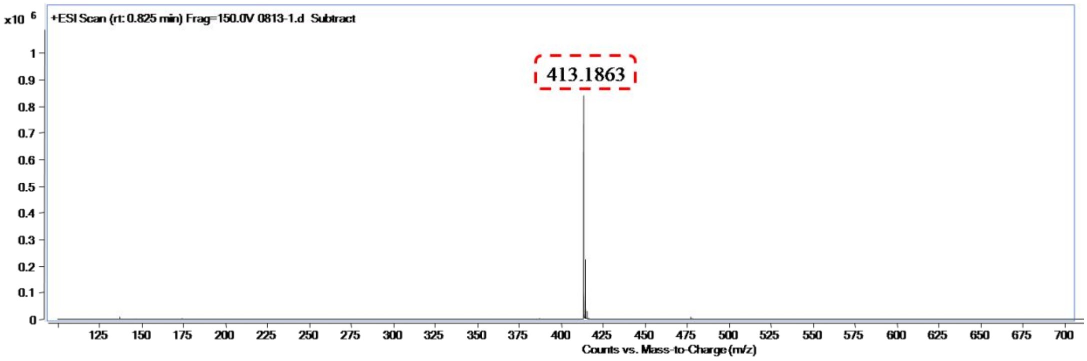

Figure S28. High resolution mass spectrum of **HC-1**.

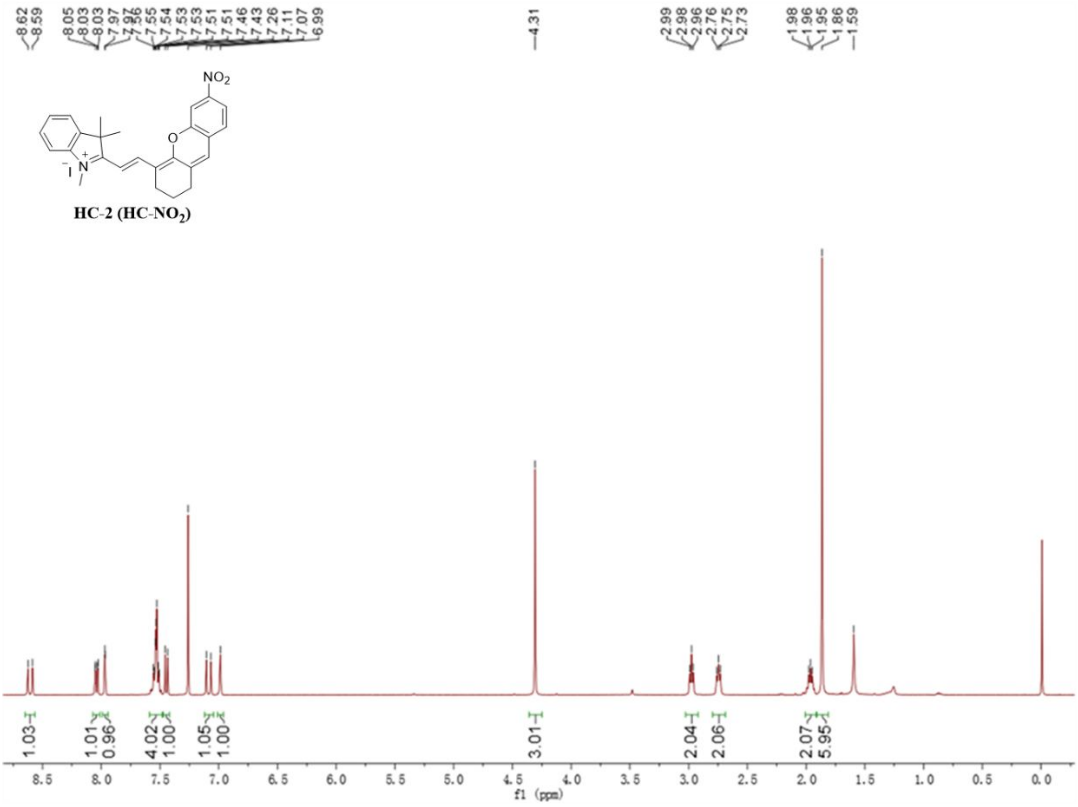

Figure S29. ^{1}H NMR spectrum of **HC-2** (**HC-NO$_2$**, CDCl$_3$).

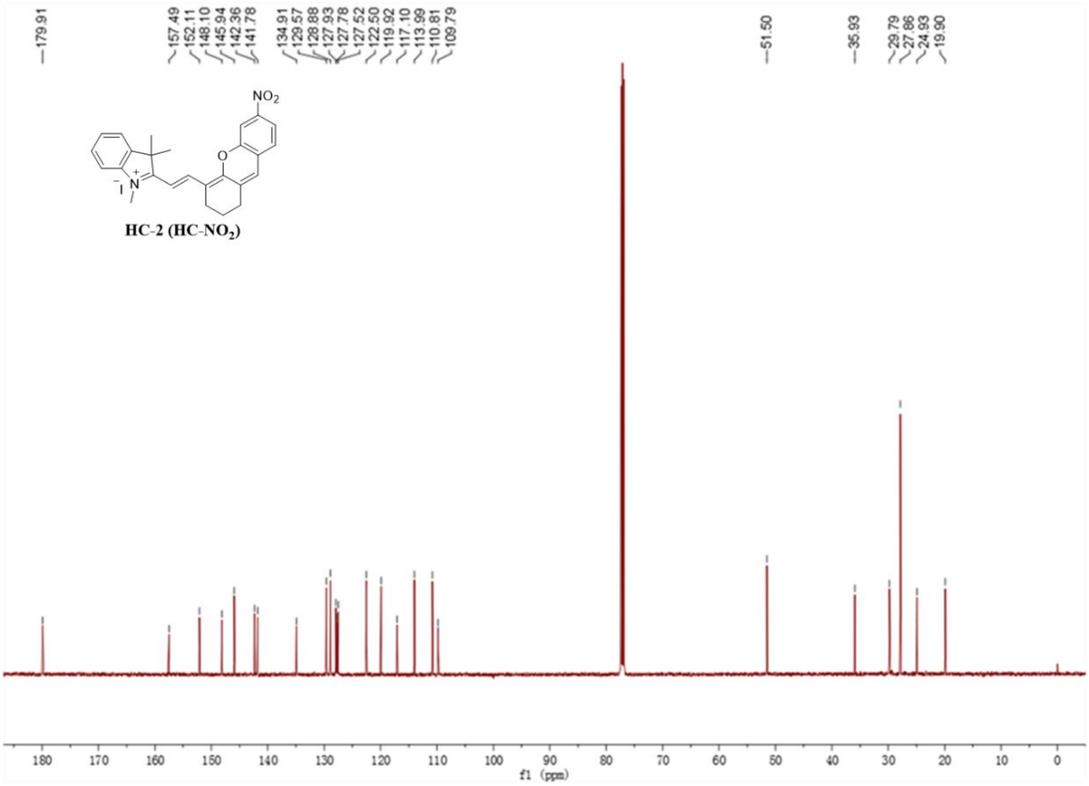

Figure S30. ^{13}CNMR spectrum of **HC-2** (**HC-NO$_2$**, CDCl$_3$).

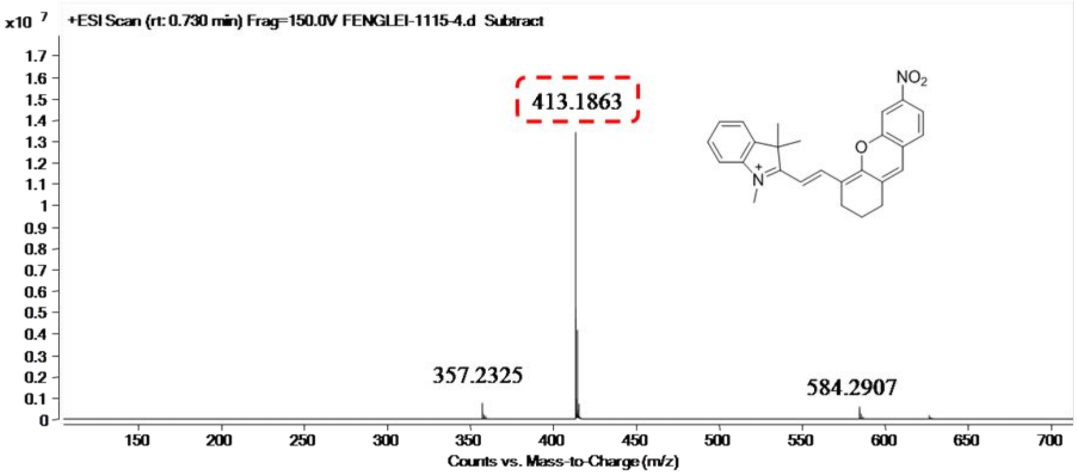

Figure S31. High resolution mass spectrum of **HC-2** (**HC-NO_2**).

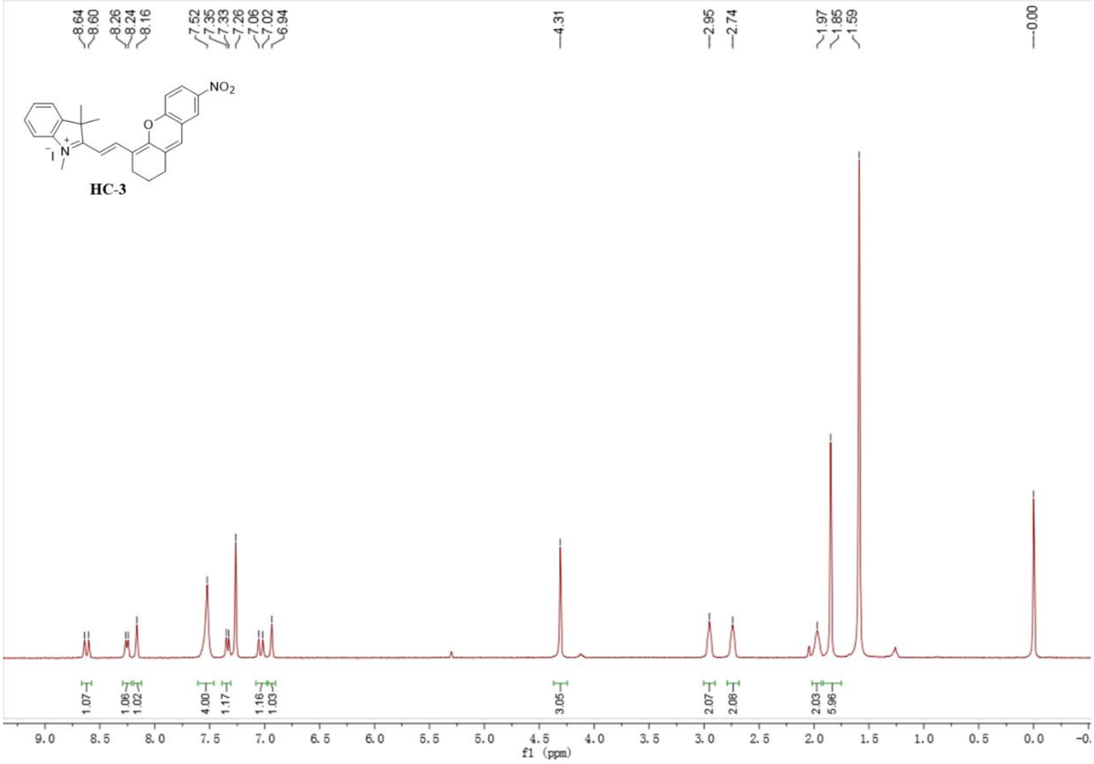

Figure S32. ^{1}H NMR spectrum of **HC-3** ($CDCl_3$).

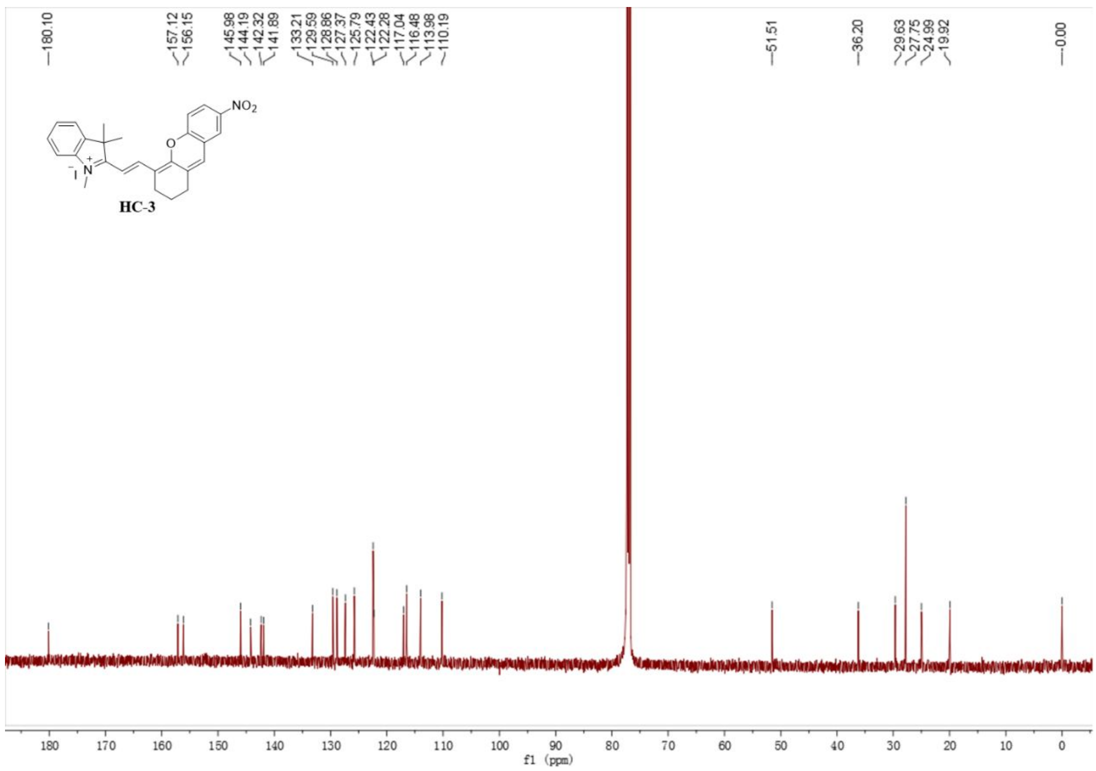

Figure S33. ^{13}CNMR spectrum of **HC-3** ($CDCl_3$).

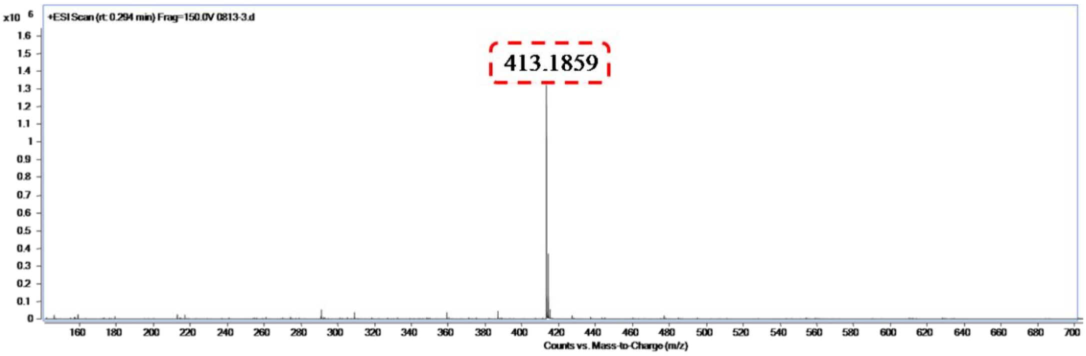

Figure S34. High resolution mass spectrum of **HC-3**.

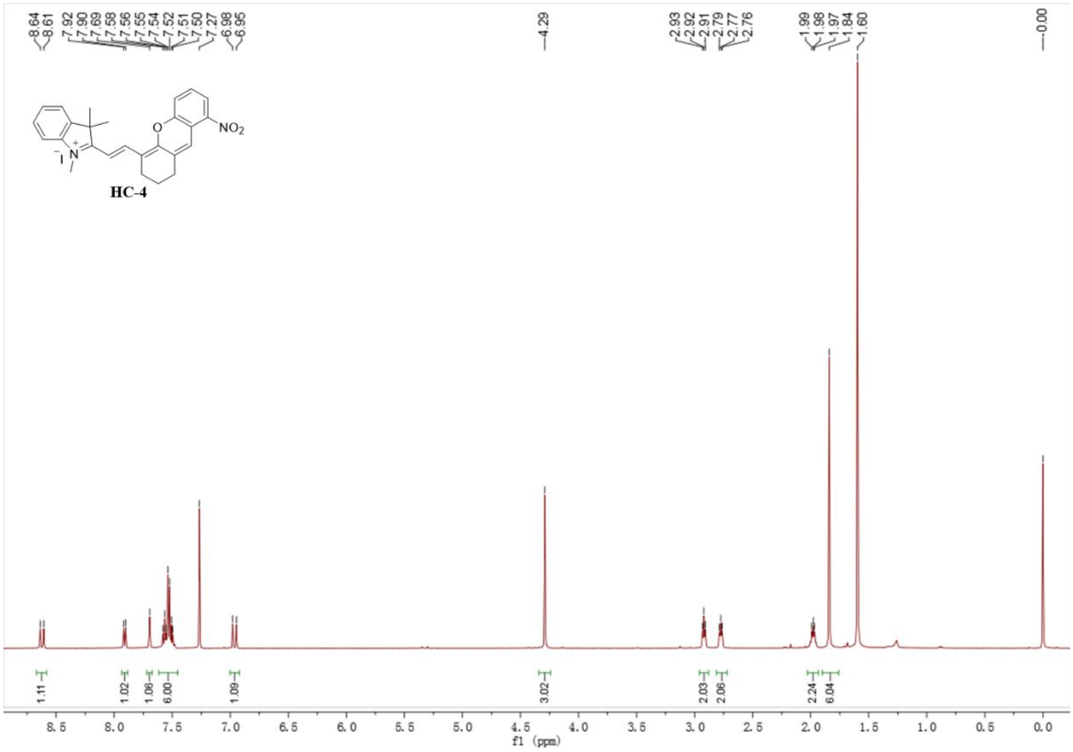

Figure S35. ^{1}H NMR spectrum of **HC-4** ($CDCl_3$).

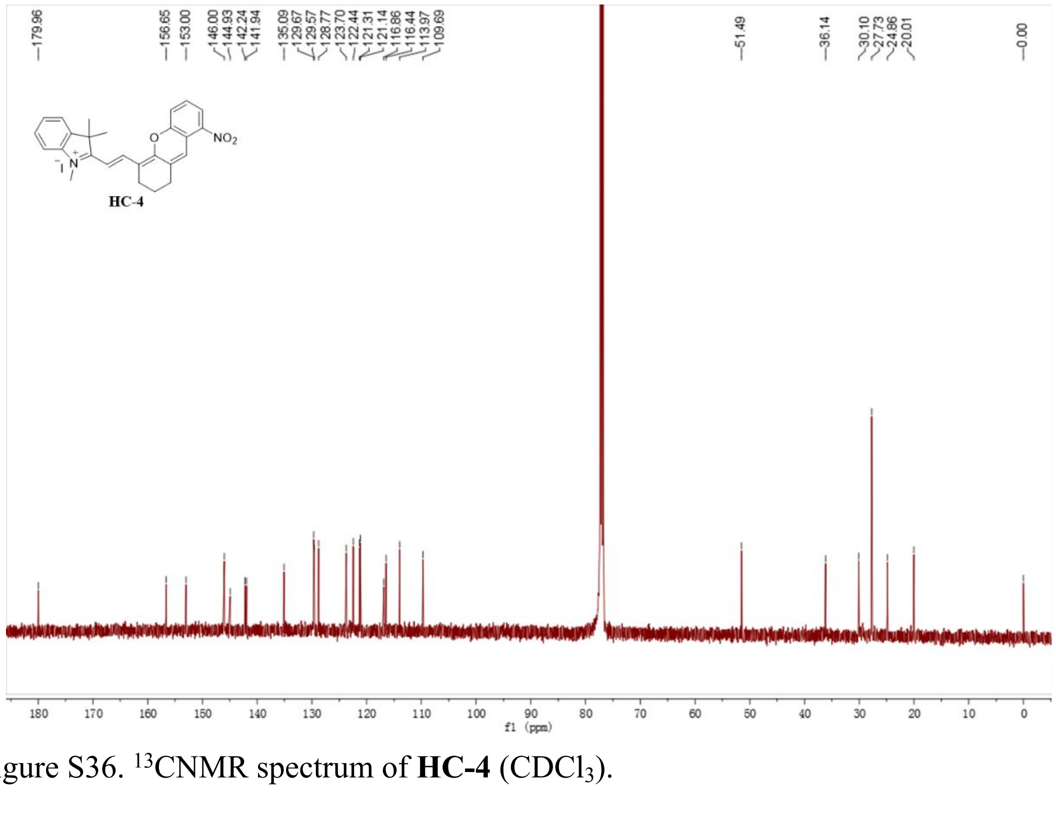

Figure S36. ^{13}CNMR spectrum of **HC-4** ($CDCl_3$).

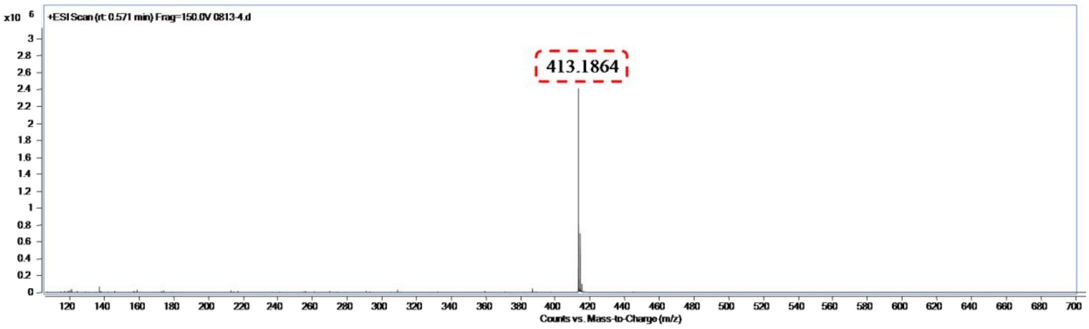

Figure S37. High resolution mass spectrum of **HC-4**.

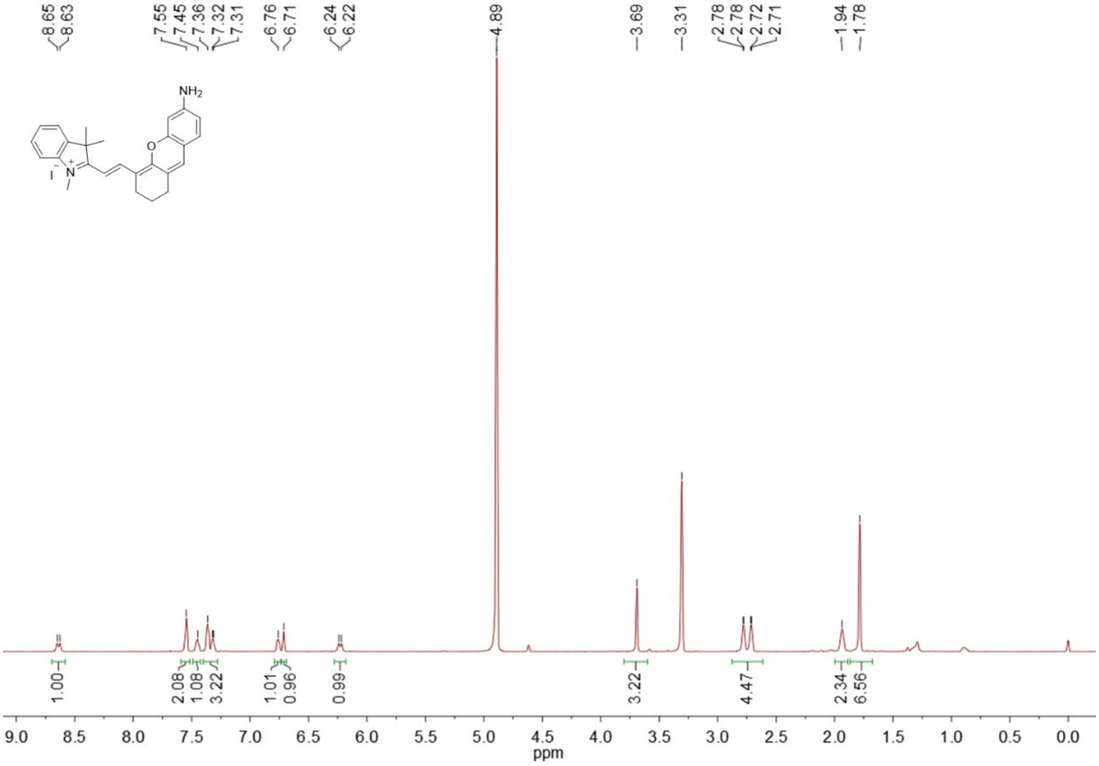

Figure S38. ^{1}H NMR spectrum of **HC-NH$_2$** (MeOD).

References

[1] Kong, F.; Liang, Z.; Luan, D.; Liu, X.; Xu, K.; Tang, B. A GSH-responsive NIR theranostic prodrug for cancer therapy and imaging [J]. *Anal. Chem*. **2016**, *88*, 6450-6456.
